# TREX (transcription/export)-NP complex exerts a dual effect on regulating polymerase activity and replication of influenza A virus

**DOI:** 10.1371/journal.ppat.1010835

**Published:** 2022-09-09

**Authors:** Lingcai Zhao, Qingzheng Liu, Jingjin Huang, Yuanlu Lu, Yongzhen Zhao, Jihui Ping

**Affiliations:** MOE Joint International Research Laboratory of Animal Health and Food Safety, Engineering Laboratory of Animal Immunity of Jiangsu Province, College of Veterinary Medicine, Nanjing Agricultural University, Nanjing, China; The Ohio State University, UNITED STATES

## Abstract

Influenza A viruses effectively hijack the intracellular "resources" to complete transcription and replication, which involve extensive interactions between the viral and host proteins. Herein, we screened the host factors, which belong to DExD/H-box protein family members, RNA-binding proteins or mitochondrial anchoring proteins, to investigate their effects on polymerase activity. We observed DDX39B and DDX39A, DEAD-box RNA-Helicases, exert a dual effect on regulating polymerase activity and replication of influenza A viruses. We further revealed that DDX39B and DDX39A interact with viral NP and NS1 proteins. Interestingly, the viral NP proteins could reverse the inhibitory effect of excess DDX39B or DDX39A on polymerase activity. Mechanistically, the TREX complex subunits, THOC1, THOC4 and CIP29, were recruited to DDX39B-DDX39A-NP complex in an ATP-dependent manner, via the interaction with DDX39B or DDX39A, followed by excess TREX-NP complexes interfere with the normal oligomerization state of NP depending on the ratio between the viral and host proteins. On the other hand, the TREX complex, an evolutionarily conserved protein complex, is responsible for the integration of several mRNA processing steps to export viral mRNA. Knockdown of TREX complex subunits significantly down-regulated viral titers and protein levels, accompanied by retention of viral mRNA in the nucleus. Taken together, screening the host factors that regulate the replication of influenza virus advances our understanding of viral pathogenesis and our findings point out a previously unclear mechanism of TREX complex function.

## Author summary

In this study, we investigated the regulation of polymerase activity by host factors associated with vRNPs (PB2_627_E, PB2_627_K, PB2_627_ domain del, PB2_627_ CON) and provided novel insights into regulatory mechanisms of DDX39B and DDX39A during viral replication. Our results demonstrated that DDX39B and its paralog DDX39A inhibited polymerase activity via forming TREX-NP complex with concomitant effects on the oligomeric state of NP proteins. Moreover, TREX complex is necessary for expression of viral proteins. Our findings provided potential therapeutic targets for dealing with IAV infection.

## Introduction

Viral pathogens including influenza, corona, and Ebola viruses can cause high morbidity and mortality with tremendous consequences for human health and the global economy. Among them, influenza virus with its wide host range is one of the most destructive pathogens that threaten the livestock industry, and the general public through annual epidemics and sporadic pandemics, such as the pandemics of 1918, 1957, 1968, and 2009.

The influenza viral polymerase complex is composed of three subunits (PA, PB1, PB2) which, together with the NP nucleoprotein, transcribes viral RNA (vRNA) into mRNA and replicates it through complementary RNA (cRNA) replication intermediates. The so-called 627 domain of PB2 forms a unique structure that protrudes from the polymerase core [1]. At amino acid position 627 of PB2, most human influenza viruses encode lysine (K), while most avian influenza viruses encode glutamic acid (E) [2]. The E-to-K mutation at PB2-627 considerably enhances the polymerase activity of avian influenza viruses at low temperature in mammalian cells, resulting in more effective virus replication in the upper respiratory tract of mammals (33–35°C) [3–5]. Collectively, the PB2-E627K mutation is now recognized as a strong determinant of host range restriction that enables avian influenza viruses to replicate efficiently in mammalian cells.

Multiple studies have sought to identify host factors related to influenza virus replication [6–9], in particular those that may account for the species-specificity of PB2-627K [10,11]. The human and avian versions of the host protein ANP32A are now known to play an important role in the host-range restriction of influenza viruses: Briefly, ANP32A from avian origin could remarkably promote the replication of avian influenza virus (PB2_627_E) [12–14]. In addition, multiple other host factors are believed to interact with the viral polymerase complex to regulate its (host-specific) activity.

In this study, we reconstructed the influenza virus polymerase complex *in vitro*, and detected the interacting proteins through immune-precipitation (IP) and mass spectrometry. We verified associated DExD/H-box protein family members, RNA-binding proteins, and mitochondrial anchoring proteins via viral microgenome polymerase activity experiments. And we first discovered that DDX39B, DDX39A and other RNA binding proteins regulate polymerase activity of influenza virus, thereby regulating replication of influenza virus. DExD/H-box helicases are further distinguished based on the amino acid sequence of the eponymous conserved helicase motif II (DEAD, DEAH, DExH and DExD helicases), which are involved in various RNA metabolic processes, including transcription, translation, RNA splicing, RNA transport, and RNA degradation [15]. There is evidence that lots of DExD/H-box helicases have also been identified as essential host factors for the replication of different viruses, suggesting that viruses "hijack" RNA helicase activities for their benefit [16,17]. Influenza virus recruits cellular RNA helicase eIF4A3 to promote viral mRNA splicing and spliced mRNA nuclear export [18]. In an interesting twist, DExD/H-box helicases also engage in anti-viral immunity, such as RIG-like helicases (RIG-I), MDA5, act as important cytosolic pattern recognition receptors for viral RNA. It is well established that viral single-stranded or short double stranded RNA bearing a 5’-triphosphate is recognized by the cytosolic RNA helicase RIG-I [19–21].

Protein-coding genes are transcribed as a pre-mRNA in the nucleus and pre-mRNA undergo several RNA processing steps. The TREX (transcription/export) complex is responsible for the integration of the several mRNA processing steps to export mRNA and is recruited to mRNA in a splicing-dependent manner [22]. The assembly of the TREX complex is dynamically associated with the ATPase cycle of DDX39B and acts as a binding platform for NXF1, and DDX39B is removed from the TREX complex during NXF1 recruitment [23–25]. Our work revealed DDX39B and DDX39A played a dual role in regulating influenza virus polymerase activity. We found that DDX39B and its paralog DDX39A play similar role in the replication of influenza virus. Further investigation of the molecular mechanism demonstrated that DDX39B and DDX39A rely on ATP binding activity to recruit TREX complex subunits THOC1, THOC4 and CIP29, and then rely on the binding of RNA to NP. Excessive DDX39B or DDX39A was not conducive to polymerase activity of influenza virus. Meanwhile, we demonstrated that DDX39B, DDX39A and other TREX complex subunits are necessary factors for expression of viral proteins, due to their involvement in mRNA transport.

## Results

### Identification and analysis of cellular binding partners of influenza virus polymerase complexes

To identify cellular interactions partners of the influenza viral polymerase complex, we reconstructed viral ribonucleoprotein (vRNP) complexes *in vitro* by transfecting human HEK293T and chicken DF-1 cells with plasmids expressing the polymerase and NP proteins of A/Anhui/1/2013 (H7N9) and a virus-like RNA encoding a reporter protein. Four different vRNPs were tested: PB2_627_E-vRNP (encoding the avian-like residue at position 627 of PB2), PB2_627_K-vRNP (encoding the human-like residue at position 627 of PB2), PB2_627_ domain del-vRNP, and PB2_627_ CON-vRNP (S1A and S1B Fig). Minireplicon assays in 293T cells confirmed that PB2_627_K-vRNP confers higher replicative ability than PB_627_E-vRNP in human cells (S1C Fig), and that the PB2 controls (PB2_627_ domain del-vRNP and PB2 CON-vRNP) are replication-incompetent.

**Fig 1:**
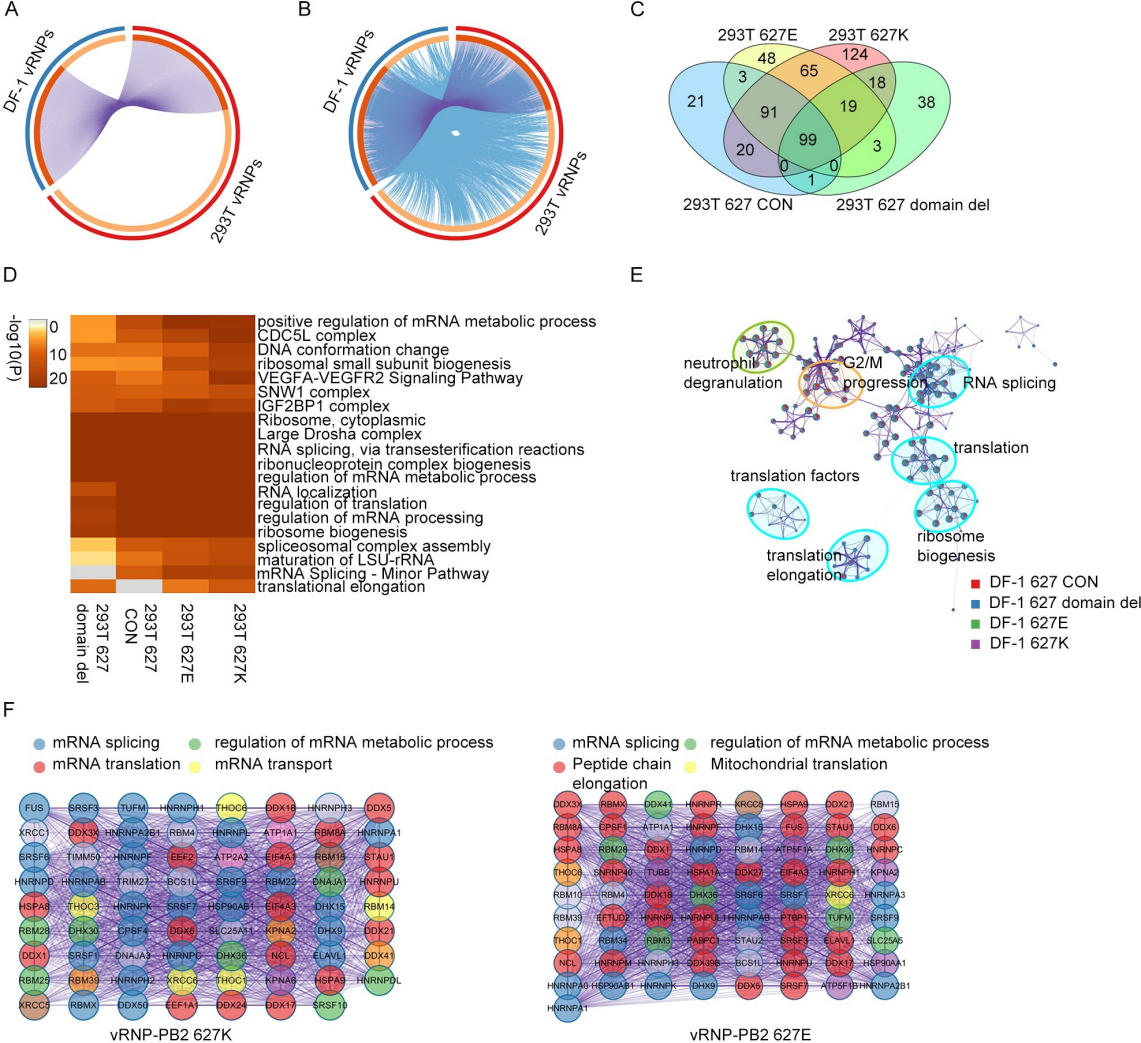
Analysis of vRNP-interaction partners and the associated cellular pathways. (A) Circos plot summarizing cellular factors that interact with influenza vRNPs in human HEK293T (red) or avian DF-1 (blue) cells. Cellular factors interacting with influenza vRNPs in both human (293T vRNPs) and avian cells (DF-1 vRNPs) are shown in dark orange and connected by purple lines; species-specific vRNP interaction partners are depicted in light orange. (B) Same as (A), but blue lines link genes that belong to the same enriched ontology term. (C) Venn diagram comparing the numbers of host factors interacting with vRNP-PB2_627_E (293T 627E), vRNP-PB2_627_K (293T 627K), vRNP-PB2_627_ domain del (293T 627 domain del), and vRNP-PB2_627_ CON (293T 627 CON) in 293T cells. The number in each sector denotes the number of cellular proteins in each category. (D) Heatmap of enriched biological processes of vRNP interaction partners. GO/KEGG-based enrichment terms were hierarchically clustered into a tree based on Kappa-statistical similarities among their gene memberships. The cells were colored by their P-values. White cells indicate lack of enrichment. Shown are the top 20 clusters. (E) Network of enriched terms represented as pie charts, where pies were color-coded based on the genes of four protein groups in DF-1 cells. Each pie sector was proportional to the number of hits originated from a different group. (F) Protein-protein interaction network of proteins associated with vRNP-PB2_627_K or vRNP-PB2_627_E. See Materials and Methods for a more detailed explanation of the pathway and process enrichment analysis.

To identify host factors interacting with vRNP complexes, the transfected cells were subjected at 48 h post-transfection to immunoprecipitation with an antibody to NP followed by LC-MS/MS mass spectrometry. In human 293T cells, 550 host proteins co-precipitated with vRNPs (Fig 1A–1C; S1 Table); in chicken DF-1 cells, vRNPs interacted with 268 host proteins (Fig 1A–1C; S1 Table). In total, 648 different host proteins bound to influenza vRNPs in human and chicken cells. Of the 550 vRNP-interacting host proteins identified in human 293T cells, 490 interacted with functional vRNP complexes (i.e., PB2_627_E-vRNP or PB2_627_K-vRNP) (S1 Table).

GO ontology analysis of vRNP-interacting host factors showed an enrichment for host factors involved in RNA splicing, ribonucleoprotein complex biogenesis, regulation of mRNA metabolic process, regulation of translation, regulation of mRNA processing, and ribosome biogenesis (Fig 1D and 1E; S2 Table). Functional vRNP complexes (i.e., PB_627_K-vRNP and PB2_627_E-vRNP) interacted with a broader set of GO ontology groups than non-functional vRNP complexes (i.e., PB2_627_ domain del-vRNP and PB2 CON-vRNP) (Fig 1E; S2 Table).

For our further analysis, we considered all human and avian vRNP-interacting host proteins after removing those that interacted with PB2_627_ CON-vRNP. For the resulting set of host proteins, we generated a protein-protein interaction network and applied the molecular complex detection (MCODE) algorithm to identify densely connected network components. Although PB2_627_E- and PB2_627_K-vRNPs showed some differences in their host protein interaction profiles (Fig 1F and S2 Table), both vRNPs interacted with a number of host proteins related to mRNA splicing (e.g., such as FUS, SRSF3, SRSF7, TUFM, HNRPA, HNRPK, HNRH1, RBMX, RBM10, RBM34, RBM3, RBM4, RBM4B and ELAVL1), with DExD/H-box proteins related to mRNA translation (such as DDX18, DDX3X, DDX5, DDX6, DDX16, DDX24, DDX17, EIF4A1, EIF4A3 and DDX39B), and with mitochondrial proteins (such as DNAJA3, SLC25A, ATP5B, ATP5C1, BCS1L and ATP5A1).

### Effect of human and avian host factors on vRNP activity

**Fig 2:**
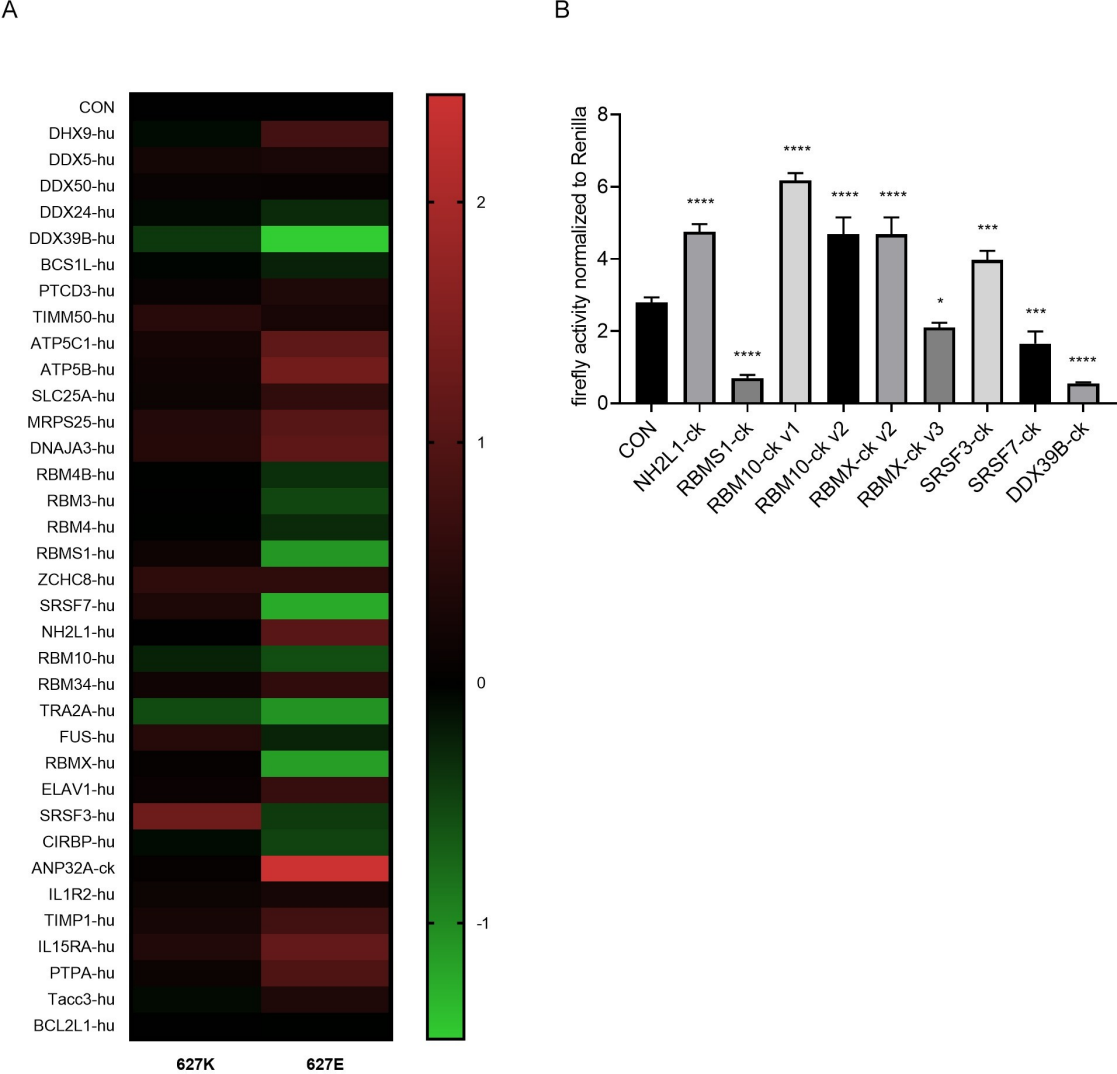
Screening host factors that regulate influenza virus polymerase activity. (A) Host proteins were selected to analyze their roles in polymerase activity (PB2_627_E or PB2_627_K) of influenza virus H7N9 (A/Anhui/1/2013). The log2 logarithmic values of the ratio of the luciferase activity transfected with the corresponding host factors and the empty pCAGGS plasmids were used as the PB2_627_E or PB2_627_K polymerase activity. (B) Host proteins from avian were selected to analyze their roles in polymerase activity (PB2_627_E) in HEK293T cells. RBM10-ck v1 and RBM10-ck v2, and RBMX-ck v2 and RBMX-ck v3 are two different transcripts of avian-derived RBM10 and RBMX, respectively. The detailed base sequences are in the attached S3 Table. Statistical differences between groups are labeled according to a one-way ANOVA followed by a Dunnett’s test. Each treatment was repeated three times in parallel. The results are presented as means ± standard deviations. *, P < 0.05; ***, P < 0.001; ****, P < 0.0001; ns, no significance. ck, chicken; hu, human.

Next, we asked if selected DExD/H-box protein family members, RNA-binding proteins, and mitochondrial anchor proteins affect influenza vRNP activity. Briefly, human 293T cells were transfected with plasmids over-expressing the indicated host protein (Fig 2). The transfected cells were co-transfected with plasmids encoding PB2_627_E- or PB2_627_K-vRNPs (Fig 2A). In the minireplicon assay (Fig 2A), overexpression of chicken ANP32A protein (which is known to increase the replicative ability of avian-type polymerase complexes in mammalian cells [14]) indeed strongly upregulated reporter protein expression by PB2_627_E-vRNP, but not by PB2_627_K-vRNP. Overexpression of several human host factors (most prominently, DDX39B, a member of the DExD/H box family of ATP-dependent RNA-helicases which is involved in mRNA splicing and nuclear export processes) reduced reporter protein expression from the influenza vRNP in the minireplicon assay. Overall, overexpression of several DExD/H-box protein family members (i.e., DDX39, DDX24), mitochondrial anchoring proteins (i.e., DNAJA3, ATP5B), and RNA-binding proteins (i.e., SRSF3, RBMX, RBM10, RBMS1, NH2L1) affected reporter protein expression from influenza vRNPs.

Previous studies established that host proteins of human or avian origin can differently affect the activity of viruses encoding PB2_627_K or PB2_627_E, respectively [26]. We, therefore, compared the effect of selected human (-hu) and chicken (-ck) RNA-binding host proteins (including splice variants of avian host proteins) on reporter protein expression in minireplicon assays (Fig 2B). Overexpression of SRSF3-hu or SRSF3-ck (serine- and arginine-rich splicing factor 3) increased the reporter protein expression levels in human cells, which is consistent with the results of our previous study [27]. On the other hand, overexpression of RBMS1-hu and RBMS1-ck reduced the reporter protein levels expressed from PB2_627_E-vRNPs (Fig 2A and 2B). This pattern indicated species-specific differences in host factor interaction with the influenza virus replication complex.

### Effect of human and avian DDX39B or DDX39A on vRNP activity

**Fig 3:**
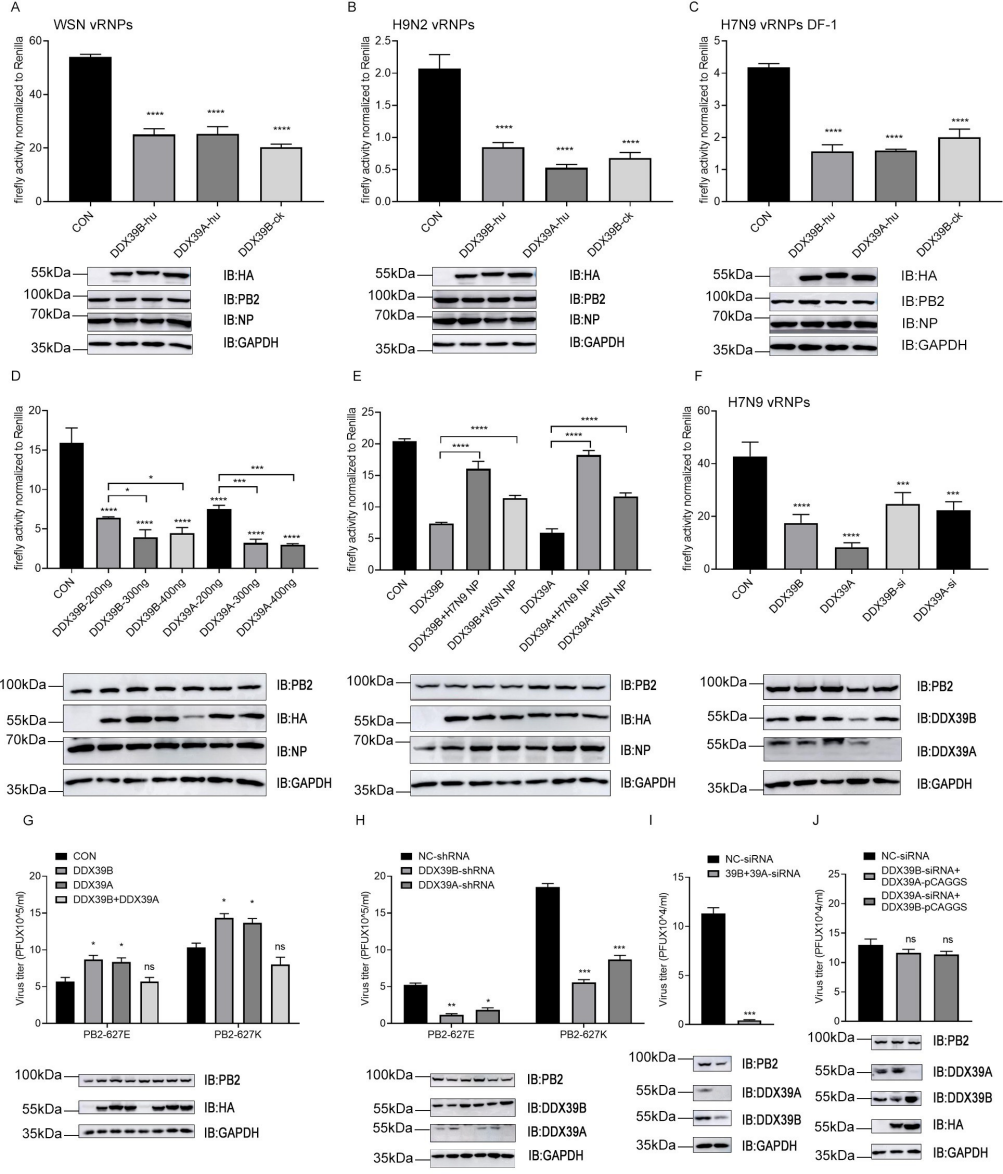
Effect of human and avian DDX39B or DDX39A on vRNP activity. (A, B and C) Polymerase genes (PB2_627_E) from either influenza virus H1N1 (A/WSN/1933) (A) or H9N2 (A/Chicken/Anhui/LH99/2017) (B), poll-Luc or pollck-Luc, RL-TK and corresponding plasmids encoding HA-DDX39B-hu, HA-DDX39A-hu, HA-DDX39B-ck or empty pCAGGS vectors were co-transfected into HEK293T cells (A and B) or DF-1 cells (C), 48h after transfection, the polymerase activity was detected. (D) Above polymerase genes (PB2_627_E), poll-Luc, RL-TK and gradually increased dose of HA-DDX39B or HA-DDX39A were co-transfected into HEK293T cells, 48h after transfection, the polymerase activity was detected. (E) Above polymerase genes (PB2_627_E), poll-Luc, RL-TK and gradually increased dose of NP of influenza virus H7N9 (A/Anhui/1/2013) or H1N1 (A/WSN/1933), 48h after transfection, the polymerase activity was detected. (F) Detecting the effect of siRNA-mediated knockdown of DDX39B or DDX39A on polymerase activity. (G) HA-DDX39B, HA-DDX39A or empty pCAGGS vectors were transfected into HEK293T cells, 24 hours after transfection, cells were infected with the recombinant influenza viruses WSN-H7 (627K) or WSN-H7 (627E) at MOI = 0.05. The supernatants were sampled at 24h post infection. (H) HEK293T cell lines with shRNA-mediated knockdown of DDX39B or DDX39A were infected with recombinant influenza viruses WSN-H7 (627K) or WSN-H7 (627E) at MOI = 0.05. The supernatants were sampled at 24h post infection. (I) HEK293T cells with siRNA-mediated knockdown of DDX39B and DDX39A, cells were infected with the recombinant influenza viruses WSN-H7 (627E) at MOI = 0.05. The supernatants were sampled at 24h post infection. (J) HA-DDX39B or HA-DDX39A was transfected into HEK293T cells with siRNA-mediated knockdown of DDX39A and DDX39B, respectively. 24 hours after transfection, cells were infected with the recombinant influenza viruses WSN-H7 (627E) at MOI = 0.05. The supernatants were sampled at 24h post infection. The protein levels were measured by western blotting and viral titers were measured by plaque assay. Statistical differences between groups are labeled according to a one-way or two-way ANOVA. The results are presented as means ± standard deviations. *, P < 0.05; **, P < 0.01; ***, P < 0.001; ****, P < 0.0001; ns, no significance. ck, chicken; hu, human.

DDX39B (also known as UAP56) is known to interact with the influenza virus NP protein [28]. However, the molecular mechanism by which DDX39B regulates viral replication remains unclear. We found that in human 293T and chicken DF-1 cells, overexpression of DDX39B-hu that was only detected in immunoprecipitates of PB2_627_E-vRNPs, DDX39A-hu (a mammalian paralog of DDX39B [29,30] which was not detected in immunoprecipitates of vRNPs (see S1 Table)), or DDX39B-ck reduced the reporter protein expression levels from vRNPs (Fig 3A–3C), consistent with the data shown in Fig 2A. Increasing amounts of DDX39B/A tended to have a stronger inhibitory on the levels of reporter protein expression from vRNPs (Fig 3D), while overexpression of NP reversed the inhibitory effect of DDX39B/A (Fig 3E). Based on these data, we reasoned that siRNA-mediated knock-down of DDX39B and DDX39A should upregulate reporter protein expression from vRNPs in minireplicon assays, but detected the opposite effect (Fig 3F). Thus, both overexpression and down-regulation of DDX39B and DDX39A reduced reporter protein expression from influenza vRNPs *in vitro*, suggesting that the level of DDX39B may be important for efficient reporter protein expression in minireplicons.

### DDX39B and DDX39A expression levels affect influenza virus replication

Next, we tested the effect of DDX39B and DDX39A overexpression on influenza virus titers in infected cells. Over-expression of either DDX39B or DDX39A hardly influenced the titers of viruses encoding PB2_627_K or PB2_627_E (Fig 3G). These data suggested that DDX39B and DDX39A may exert effects in multiple processes of virus replication, or the virus adopts certain strategies to suppress their inhibitory effects.

shRNA-mediated down-regulation of DDX39B or DDX39A reduced virus titers in infected cells (Fig 3H); this inhibitory effect was further enhanced when both DDX39B and DDX39A were down-regulated (Fig 3I). Moreover, the siRNA-mediated reduction of virus titers was offset by overexpression of the DDX39 paralog (Fig 3J). Collectively, our data indicate that DDX39B and DDX39A are essential host factors for efficient viral replication, and their inhibitory effects on polymerase activity may depend on the ratio between viral and host proteins.

### DDX39B and DDX39A expression levels and intracellular localization are not affected by influenza virus infection

**Fig 4:**
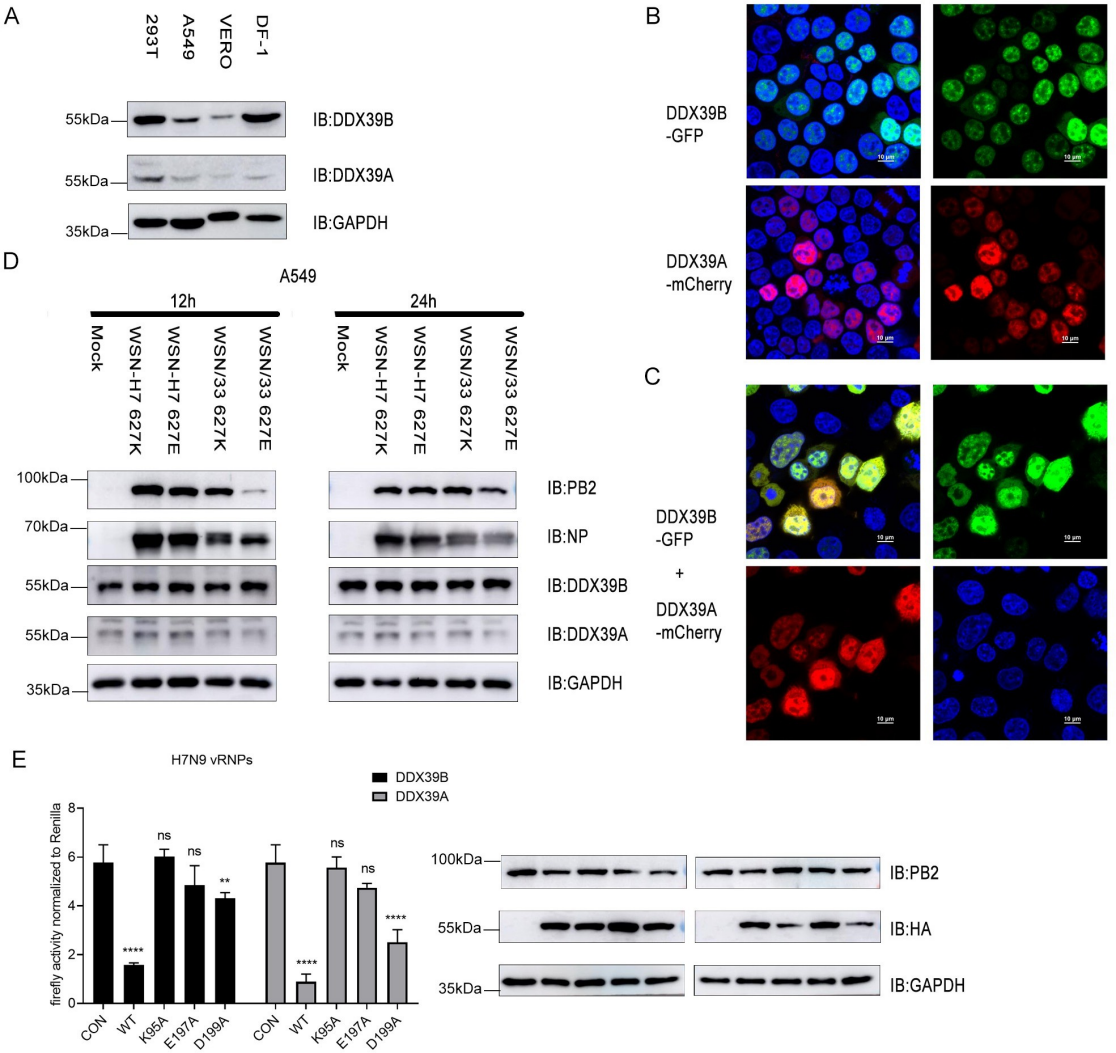
DDX39B and DDX39A inhibit polymerase activity depending on ATP binding and hydrolytic activity. (A) The protein levels of DDX39B and DDX39A in HEK293T, A549, Vero and DF-1 cells were measured by western blotting. (B) DDX39B-GFP or DDX39A-mCherry was transfected into HEK293T cells, 24 hours after transfection, the cells were then fixed, permeabilized. Cells were stained with the 4, 6-diamidino-2-phenylindole (DAPI) and were examined via fluorescence microscopy. (C) DDX39B-GFP and DDX39A-mCherry were co-transfected into HEK293T cells, 24 hours after transfection, the cells were treated the same as in (B). (D) A549 cells were infected with influenza virus WSN/1933 627K, WSN/1933 627E or recombinant influenza virus WSN-H7 (627K), WSN-H7 (627E) at MOI = 0.5. At 12h, 24h after infection, the cells were lysed and protein levels were measured by western blotting. (E) Polymerase genes (PB2_627_E), poll-Luc, RL-TK and corresponding HA-tagged mutants of DDX39B or DDX39A or empty pCAGGS vectors were co-transfected into HEK293T cells, 48h after transfection, the polymerase activity is detected. Statistical differences between groups are labeled according to two-way ANOVA. The results are presented as means ± standard deviations. **, P < 0.01; ****, P < 0.0001; ns, no significance.

To identify the molecular mechanisms through which DDX39B and DDX39A affect the influenza virus live cycle, we first assessed their expression levels and intracellular localization. In four different mammalian and avian cell lines, the expression levels of DDX39B and DDX39A vary widely (Fig 4A), in line with RNA-seq tissue data from public databases [31]. GFP- and mCherry-tagged DDX39B and DDX39A fusion proteins, respectively, are primarily expressed in the nucleus (Figs 4B, 4C, and S2A). Infection of human A549 cells with four different influenza viruses expressing PB2_627_K or PB2_627_E had no significant effect on the expression levels (Fig 4D) and intracellular localization (S2B and S2C Fig) of DDX39B and DDX39A.

### DDX39B and DDX39A mutants no longer reduce influenza virus polymerase activity in minireplicon assays

DDX39B is an essential splicing factor which is required for the U2 snRNP-branchpoint interaction during pre-spliceosome assembly, and its ATP-binding, ATPase, and double-stranded (ds) RNA unwinding/helicase activities are essential for *in vitro* pre-mRNA splicing [32]. To determine if these functions are important for the regulation of influenza virus polymerase activity, we constructed DDX39B mutants lacking ATP-binding (K95A), ATPase activity (E197A), or dsRNA unwinding/helicase activity (D199A) (S3 Fig) [32–34]. Compared with wild-type DDX39B and DDX39A, the K95A and E197A mutants lost the ability to reduce reporter protein expression from influenza vRNPs in minireplicon assays (Fig 4E), demonstrating that the ATP-binding and ATPase activities of DDX39B and DDX39A are needed to suppress reporter protein expression in minireplicon assays.

### DDX39B and DDX39A interact with the influenza virus NP and NS1 proteins

**Fig 5:**
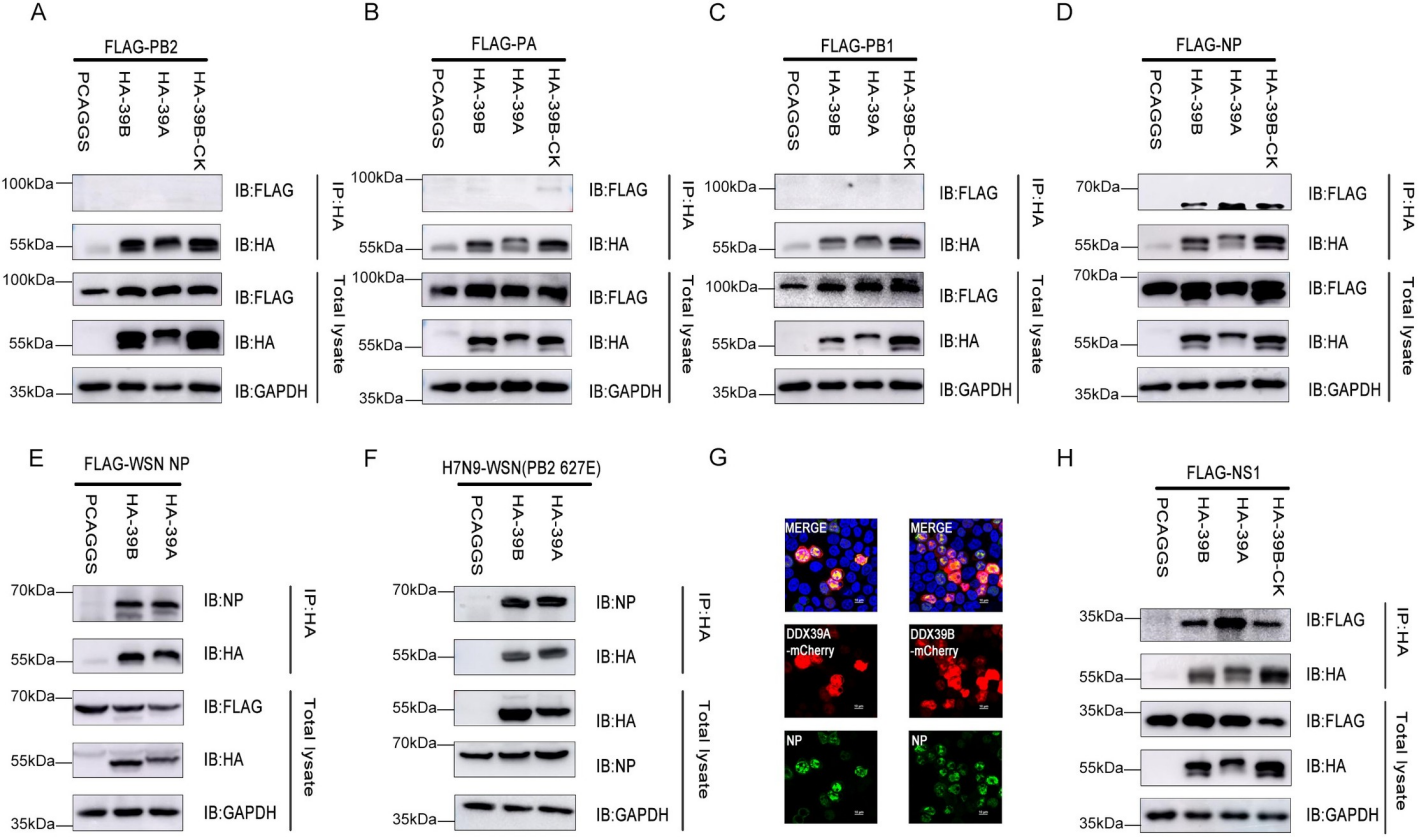
DDX39B and DDX39A interact with NP and NS1 protein. (A, B, C, D and E) HEK293T cells were co-transfected with HA-DDX39B, HA-DDX39A or HA-DDX39B-ck and FlAG-PB2 (A), FlAG-PA (B), FlAG-PB1 (C), FlAG-NP (D) of influenza virus A/Anhui/1/2013 (H7N9) or FlAG-NP of influenza virus A/WSN/1933 (H1N1) (E), at 48h after transfection, the cells were lysed, followed by co-IP with anti-HA mouse Mab and western blotting using anti-FlAG mouse Mab and anti-HA mouse MAb. (F) HEK293T cells were transfected with HA-DDX39B or HA-DDX39A, at 24h after transfection, the cells were infected with recombinant influenza virus WSN-H7 627E at MOI = 1. At 24h after infection, the cells were lysed, followed by co-IP with anti-HA mouse Mab and western blotting using anti-NP mouse Mab and anti-HA mouse MAb. (G) DDX39B-mCherry or DDX39A-mCherry together with NP were transfected into HEK293T cells, 24 hours after transfection, the cells were fixed and examined via fluorescence microscopy. (H) HEK293T cells were co-transfected with HA-DDX39B, HA-DDX39A or HA-DDX39B-ck and FLAG-NS1 of influenza virus A/WSN/1933 (H1N1), at 48h after transfection, the cells were lysed, followed by co-IP with anti-HA mouse Mab and western blotting using anti-FlAG mouse Mab and anti-HA mouse MAb.

DDX39B co-precipitated with vRNP complexes (S1 Table). DDX39B is known to interact with influenza virus NP, but has not been tested for interaction with the other components of the viral replication complex. We, therefore, performed co-immunoprecipitation studies with the three viral polymerase proteins and NP, but only the latter interacted with the host factors (Fig 5A–5E). The interaction of DDX39B and DDX39A with NP was also detected in the context of virus-infected cells (Fig 5F), in line with the co-localization of these proteins in virus-infected cells (Fig 5G).

We also tested the remaining influenza viral proteins for an interaction with DDX39B or DDX39A and found that the viral interferon-antagonist NS1 protein co-precipitated with both of these host factors (Fig 5H), consistent with a report that these proteins co-localize [35]. However, overexpression of NS1 had no significant effect on DDX39B’s ability to down-regulate reporter protein expression from vRNPs in minireplicon assays (S4A Fig). The other viral proteins tested (i.e., the M1 matrix protein, the M2 ion channel protein, and the NS2/NEP nuclear export protein) did not interact with DDX39B and DDX39A (S4B–S4D Fig) and their overexpression did not affect the DDX39A-mediated reduction of reporter protein expression from vRNPs in minireplicon assays (S4A Fig).

### Effects of DDX39B and DDX39A on polymerase activity depend on ATP to recruit TREX complex subunits THOC1, THOC4 and CIP29

**Fig 6:**
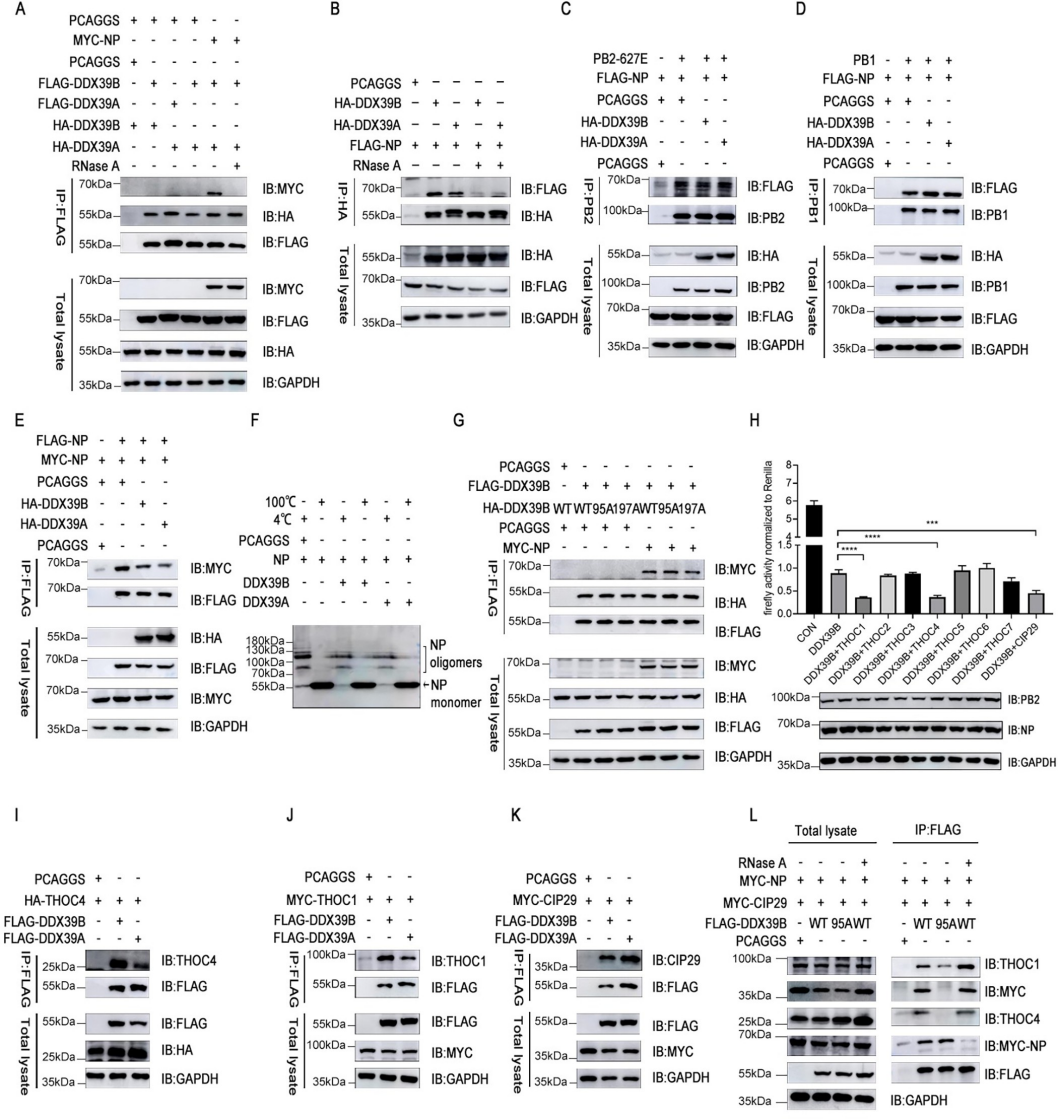
Effects of DDX39B and DDX39A on polymerase activity depend on ATP to recruit TREX complex subunits THOC1, THOC4 and CIP29. (A) HEK293T cells were co-transfected with HA-DDX39B or FLAG-DDX39B, HA-DDX39A or FLAG-DDX39A, MYC-NP or empty pCAGGS vectors, at 48h after transfection, the cells were lysed treated with or without RNase A, followed by co-IP with anti-FLAG mouse Mab. (B) HEK293T cells were co-transfected with FLAG-NP and HA-DDX39B, HA-DDX39A or empty pCAGGS vectors, at 48h after transfection, the cells were lysed and treated with or without RNase A, followed by co-IP with anti-HA mouse Mab. (C) HEK293T cells were co-transfected with FLAG-NP and PB2 together with HA-DDX39B, HA-DDX39A or empty pCAGGS vectors, at 48h after transfection, the cells were lysed, followed by co-IP with anti-PB2 mouse Mab. (D) HEK293T cells were co-transfected with FLAG-NP and PB1 together with HA-DDX39B, HA-DDX39A or empty pCAGGS vectors, at 48h after transfection, the cells were lysed, followed by co-IP with anti-PB1 mouse Mab. (E) HEK293T cells were co-transfected with FlAG-NP and MYC-NP together with HA-DDX39B, HA-DDX39A or empty pCAGGS vectors, at 48h after transfection, the cells were lysed, followed by co-IP with anti-FLAG mouse Mab. (F) HEK293T cells were co-transfected with NP and HA-DDX39B or HA-DDX39A, at 24h after transfection, the cells were lysed and cell lysates were divided into 2 portions: stored at 4°C or heated at 100°C for 5 min. Reducing SDS/PAGE with 5% acrylamide in the stacking gel and 10% in the running gel was carried out at 4°C and at low current. (G) HEK293T cells were co-transfected with FlAG-DDX39B and HA-DDX39B (WT), HA-DDX39B (WT), HA-DDX39B (95A), HA-DDX39B (197A) or HA-DDX39B (199A) together with MYC-NP or empty pCAGGS vectors, at 48h after transfection, the cells were lysed, followed by co-IP with anti-FLAG mouse Mab. (H) Polymerase genes (PB2_627_E), poll-Luc, RL-TK and HA-DDX39B together with plasmids encoding THOC1, THOC2, THOC3, THOC4, THOC5, THOC6, THOC7, CIP29 or empty pCAGGS vectors were co-transfected into HEK293T cells, 48h after transfection, the polymerase activity was detected. (I, J and K) HEK293T cells were co-transfected with MYC-THOC1 (I), HA-THOC4 (J) or MYC-CIP29 (K) and FLAG-DDX39B or FLAG-DDX39A, at 48h after transfection, the cells were lysed, followed by co-IP with anti-FLAG mouse Mab. (L) HEK293T cells were transfected with FLAG-DDX39B (WT), FLAG-DDX39B (95A) or empty pCAGGS vectors, at 24h after transfection, the cells were infected with recombinant influenza viruses WSN-H7 (627E) (MOI = 1) and cultured for 12 h, then cells were lysed and treated with or without RNase A, followed by co-IP with anti-FLAG mouse Mab.

Based on our data, we speculated that DDX39B and DDX39A may regulate influenza vRNP activity through their interaction with NP. Co-immunoprecipitation studies with differently tagged versions of DDX39B and DDX39A further indicated that DDX39B, DDX39A, and NP form trimeric complex (Fig 6A). Since both DDX39B and NP have RNA binding properties [36,37], we also tested the role of RNA in the DDX39B, DDXA39A, and NP interaction and found that RNase A treatment of cell lysates abolished the interaction between NP and DDX39B/DDX39A (Fig 6A and 6B), demonstrating a crucial role for RNA in the interaction of NP with DDX39B and DDX39A, which is different from the experiments of prokaryotic proteins in vitro [28,38]. It was reported NP served as a switch between transcription and replication and NP interacts with PB1 and PB2 but not PA. We observed that overexpression of DDX39B had no obvious effect on the interaction between NP and PB1 or PB2 (Fig 6C and 6D). We found that excessive DDX39B and DDX39A slightly affected the interaction of NP protein itself, thereby affecting oligomerization state of NP (Fig 6E and 6F). Of course, the mechanism of action of DDX39B and DDX39A in the process of NP oligomerization needs to be further elucidated in future work.

The inhibitory effect of DDX39B and DDX39A on reporter protein expression from influenza vRNPs in minireplicon assays depended on the ATP-binding and hydrolytic activity of DDX39B (see Fig 4E). However, DDX39B and DDX39A mutants lacking ATP-binding and hydrolytic activity still interacted with NP (Fig 6G), indicating that other proteins may be involved in the interaction of DDX39B-NP. DDX39B promotes pre-spliceosome assembly by interacting with the splicing factor U2AF2 in an ATP-dependent manner. Moreover, DDX39B forms complexes with the RNA-splicing and -export protein SRRM1/SRm160 [39], and we found that another pre-mRNA splicing factor, SRSF7, may regulate influenza virus polymerase activity in minireplicon assays (Fig 2). Thus, we speculated that the ATP-dependent formation of pre-spliceosomes may be important for efficient influenza virus replication. DDX39B co-immunoprecipitated with the splicing factor U2AF2, but not with the pre-mRNA splicing factor SRSF7 (S5A Fig). However, in minireplicon assays, overexpression of U2AF2, SRSF7, and SRRM1 had no significant effect on DDX39B’s regulation of polymerase activity, although SRSF7 itself reduced polymerase activity (S5B Fig).

In addition to participating in the assembly of the spliceosome, DDX39B forms a transcription export (TREX) complex with ALYREF/THOC4, CIP29/SARNP and the THO subcomplex, which comprises THOC1/HPR1, THOC2/hTHO2, THOC3/hTEX1, THOC5/fSAP79, THOC6/fSAP35 and THOC7/fSAP24 [40,41]. TREX complexes play an important role in RNA transcription, processing, and nuclear export. Overexpression of THOC1 and CIP29 protein reduced reporter protein expression levels from influenza vRNPs in minireplicon assays, while the other factors tested had no effect (S5C Fig). Moreover, over-expression of THOC1, THOC4, and CIP29 together with DDX39B (Fig 6H) or DDX39A (S5D Fig) inhibited reporter protein expression from vRNPs more strongly than did over-expression of DDX39B or DDX39A alone. Co-immunoprecipitation studies also showed an interaction of DDX39B and DDX39A with THOC1, THOC4, and CIP29 (Fig 6I–6K). Since the DDX39B and DDX39A-mediated regulation of vRNP activity depends on the ATP-binding and hydrolysis activity of the two host factors (see Fig 4E), we next performed co-immunoprecipitation studies with DDX39B mutants lacking the ATP-binding activity. Co-immunoprecipitation experiments revealed the formation of DDX39B/THOC1/THOC4/CIP29/NP complexes during infection (Fig 6L); as found earlier, RNase A treatment resulted in the loss of NP from the complex. Interestingly, CIP29 bound to wild-type DDX39B, but not to the DDX39B-95A mutant, and DDX39B mutant lacking the ATP-binding activity has significantly reduced affinity for THOC1 and THOC4. We further found that silencing THOC1, THOC4 or CIP29 blocked the down-regulation of polymerase activity by overexpressing DDX39B, which implied TREX complex is directly involved in the DDX39B-NP mediated regulation of polymerase activity (S5E and S5F Fig). Next, we exogenously overexpressed DDX39B or its mutant (95A) with THOC1, THOC4 or CIP29 in HEK293T cells, further elucidating the ATP-binding activity-dependent recruitment of TREX complex subunits by DDX39B (S6A–S6C Fig). Immunofluorescence experiments demonstrated DDX39B and/or DDX39A colocalized extensively with THOC1/THOC4/CIP29 and NP proteins in cells (S6D and S6E Fig). Thus, the influenza virus NP protein interacts with the TREX complex, which exerts a dual effect on regulating polymerase activity.

### TREX complex facilitate influenza virus mRNA nuclear export

**Fig 7:**
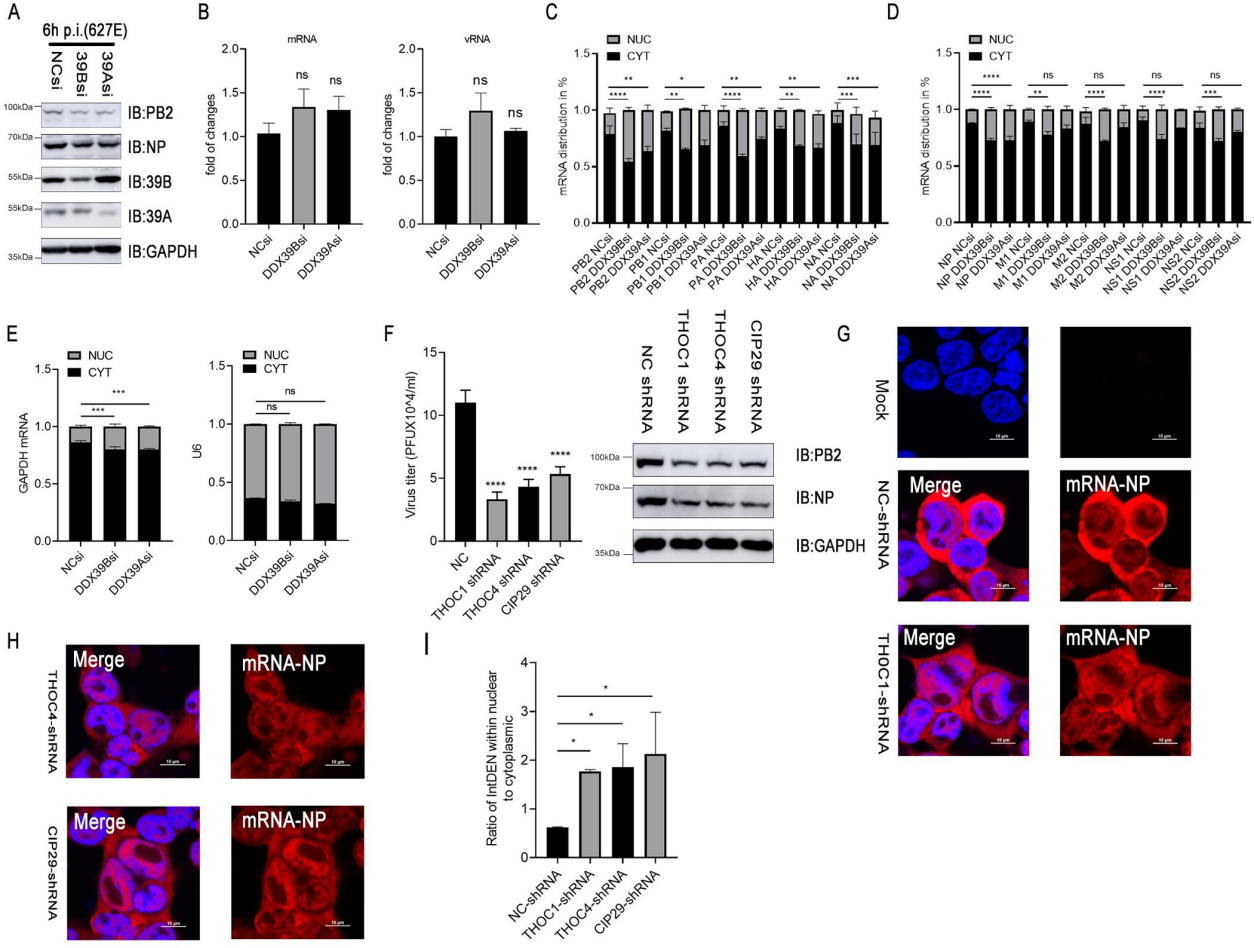
TREX complex are necessary for mRNA nuclear export during the replication of influenza virus. (A and B) HEK293T cells were transfected with the indicated siRNA for 24 h and were infected with the recombinant influenza viruses WSN-H7 (627E) at MOI = 2 without TPCK-trypsin, followed by western blotting to measure the protein levels (A) and qPCR to detect PB2 mRNA or PB2 vRNA at 3h after infection (B). GAPDH served as an internal control. (C, D and E) HEK293T cells were transfected with the indicated siRNA for 24 h and infected with the recombinant influenza viruses WSN-H7 (627E) at MOI = 2. Cells are subjected to nuclear-cytoplasmic separation at 12h after infection and measurements of the viral mRNAs (C and D) and GAPDH, U6 (E) served as an internal control. (F) HEK293T cell lines with shRNA-mediated knockdown of THOC1, THOC4 or CIP29 were infected with recombinant influenza viruses WSN-H7 (627E) at MOI = 0.05. The supernatants were sampled at 24h post infection. The virus titers were determined by plaque assay and protein levels were detected by western blotting. (G and H) HEK293T cell lines with shRNA-mediated knockdown of THOC1, THOC4 or CIP29 were infected with the recombinant influenza viruses WSN-H7 (627E) at MOI = 2. Cells were stained with cy3-labeled NP mRNA probes at 12h after infection, followed by FISH analysis. (I) Quantification of fluorescence signal was performed in ImageJ. Statistical differences between groups are labeled according to one-way ANOVA. The results are presented as means ± standard deviations. *, P < 0.05; **, P < 0.01; ***, P < 0.001; ****, P < 0.0001; ns, no significance.

Our data indicated that DDX39B and DDX39A are part of the TREX complex; they may thus regulate viral protein expression levels by affecting mRNA transport. Next, we analyzed the consequences of DDX39B or DDX39A down-regulation during influenza virus infection; the viral protein levels were assessed six hours after infection and in the absence of TPCK-trypsin, thereby preventing multiple rounds of virus replication. Down-regulation of DDX39B or DDX39A decreased the levels of several viral proteins (Fig 7A). On the other hand, down-regulation of DDX39B or DDX39A did not reduce the levels of viral RNAs and viral mRNAs in infected cells (Fig 7B), indicating that the inhibitory effect of DDX39B and DDX39A occurred at the post-transcriptional level. Since the TREX complex plays a role in mRNA export from the nucleus, we next compared the ratios of nuclear and cytoplasmic viral mRNAs in infected cells treated with siRNA to DDX39B or DDX39A, or treated with a control siRNA. Down-regulation of DDX39B or DDX39A increased the relative amounts of nuclear viral mRNAs compared to cytoplasmic viral mRNAs (Fig 7C and 7D), although some of the differences were small. We also tested two host mRNAs and found no difference for U6 mRNA, but a slight increase in nuclear GAPDH mRNA amounts upon treatment with siRNA to DDX39B/DDX39A (Fig 7E). Collectively, the data suggested that DDX39B and DDX39A are involved in the nucleocytoplasmic transport of influenza viral mRNAs. To further analyze the role of TREX complex in influenza virus replication, we constructed THOC1, THOC4 or CIP29 knockdown cell lines (S5E Fig). We found that knocking down THOC1, THOC4 or CIP29 would inhibit influenza virus replication and protein levels (Fig 7F). The Fluorescence in situ hybridization (FISH) experiment further proved that TREX complex played an important role in viral mRNA nuclear and cytoplasmic transport (Fig 7G–7I). Taken together, the above results demonstrated that DDX39B and DDX39A cooperated with subunits of the TREX complex to participate in nuclear and cytoplasmic transport of viral mRNA.

## Discussion

The global spread of viral pathogens, such as IAV and SARS-CoV-2, led to major economic and health challenges worldwide [42–44]. Revealing host genes essential for infection by IAV can provide insights into the virus pathogenesis, and facilitate the development of novel therapeutics. Influenza virus RNA polymerase and NP protein together constitute viral ribonucleoprotein complexes (vRNPs), thus serving as the smallest structural unit of viral genome transcription and replication. In this study, we sought to obtain proteins associated with reconstructed four vRNPs mutants (PB2_627_E, PB2_627_K, PB2_627_ domain del, PB2_627_ CON) in HEK293T cells and DF-1 cells via co-immunoprecipitation assay and LC-MS/MS mass spectrometry. To gain a more complete understanding of coprecipitated host proteins, we analyzed enriched gene ontology and overrepresented pathway, which indicated RNA splicing, ribonucleoprotein complex biogenesis, regulation of mRNA metabolic process, regulation of translation, regulation of mRNA processing and ribosome biogenesis are important in the replication and transcription of influenza viruses. Subsequently, we generated a protein-protein interaction network. Of note, the phenomenon that many host proteins identified in the interconnected network are bystanders, or perhaps interacting with NP alone but not vRNPs, should be considered. Our results showed that many DExD/H-box protein family members (DDX39B), mitochondrial anchoring proteins (ATP5C1, ATP5B) and RNA-binding proteins (RBMS1, SRSF3, and SRSF7) regulate polymerase activity of influenza viruses. Interestingly, RBMS1 and SRSF7 from human may exist as inhibitory factors for avian influenza virus, while RBM10 and SRSF3 as positive cofactors. Of course, further work is needed to better understand the molecular mechanism of species-specific differences. Importantly, we discovered that DDX39B and its paralog DDX39A, which is not expressed in avian-derived cells, markedly inhibited the polymerase activity, especially carrying PB2_627_E.

DDX39B and DDX39A, members of the DEAD-box family of RNA-dependent ATPases that mediate ATP hydrolysis during pre-mRNA splicing, belong to a cluster of genes localized in the vicinity of the genes encoding tumor necrosis factor alpha and tumor necrosis factor beta, which are within the human major histocompatibility complex class III region. The involvement of DDX39B and DDX39A in influenza virus replication has been reported, however, the understanding on the mechanism by which they exert effects remains limited. Our results showed that silencing DDX39B or DDX39A significantly inhibited the polymerase activity and replication of influenza viruses. DDX39B and DDX39A inhibited polymerase activity in a dose-dependent manner, and surprisingly, an increase in NP protein, but not NS1, reversed such inhibitory effects, although DDX39B and DDX39A could interact with viral NP and NS1 proteins. The above experimental results suggest that the versatile DDX39B and DDX39A may play roles in multiple cellular biological processes and participate in multiple steps in the replication cycle of influenza viruses, which is different from previous understanding. Next, we revealed that DDX39B and DDX39A regulated the polymerase activity of influenza virus depending on ATP binding activity and ATP hydrolysis activity, whereas the DDX39B-NP interaction dose not. Our results further reveal that DDX39B-DDX39A-NP complexes are formed in an RNA-dependent manner, with the consequent effect that accumulation of excess DDX39B or DDX39A interferes with the oligomeric state of free NP proteins, which explains that DDX39B and DDX39A inhibit polymerase activity carrying PB2_627_E, lower polymerase activity and producing relatively less vRNPs, to a greater extent.

**Fig 8:**
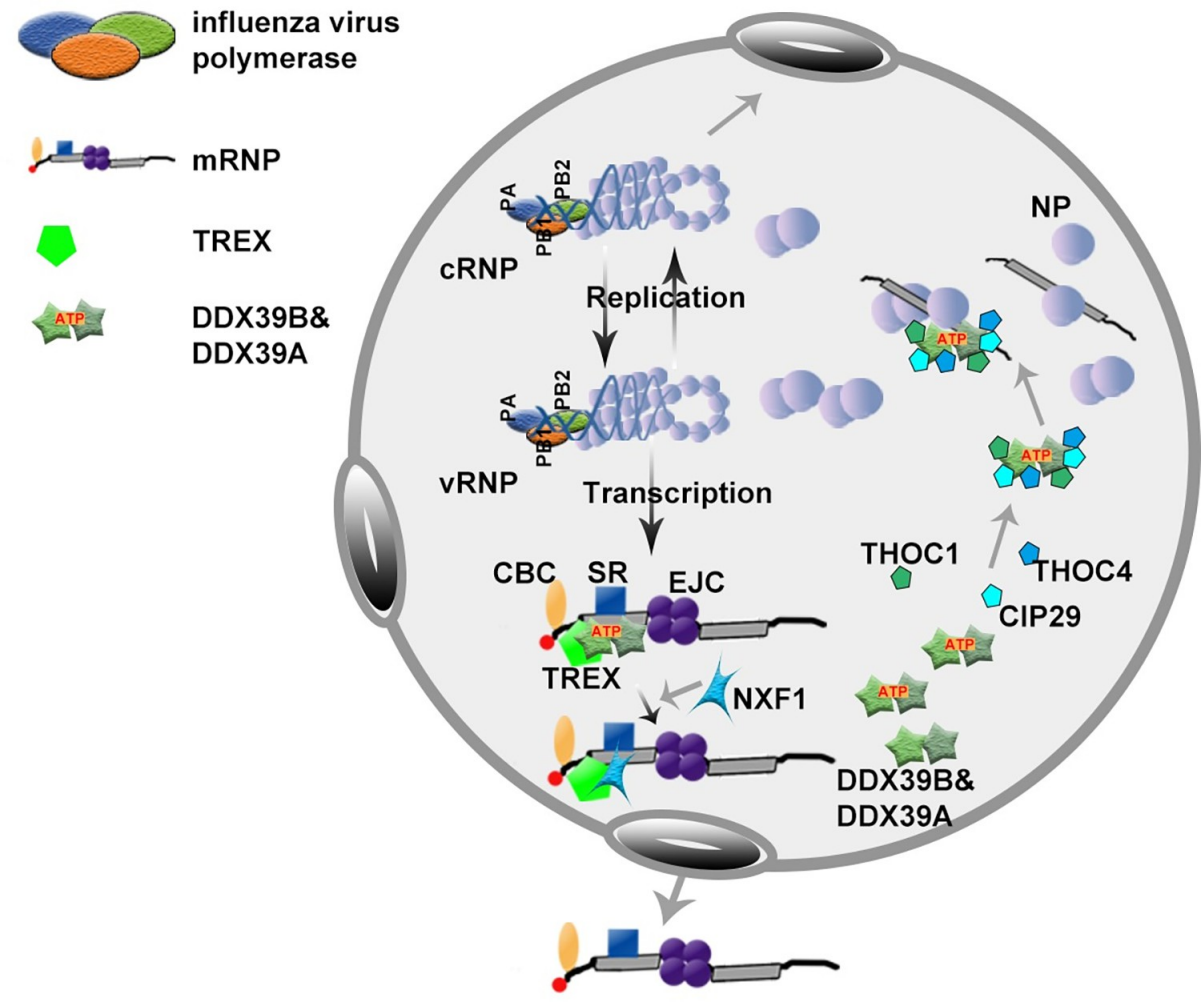
Schematic model of TREX (transcription/export)-NP complex involved in replication and transcription of influenza A virus. The TREX complex subunits, THOC1, THOC4 and CIP29, were recruited to DDX39B-DDX39A-NP complex in an ATP-dependent manner via the interaction with DDX39B or DDX39A. However, excess TREX-NP complexes interfere with the normal oligomerization state of NP depending on the ratio between the viral and host proteins. Interestingly, NP protein, but not NS1, reversed such inhibitory effects. On the other hand, DDX39B and DDX39A cooperated with subunits of the TREX complex to participate in nuclear and cytoplasmic transport of viral mRNA.

RNA polymerase II (Pol II) transcribes most genes as RNA precursors that mature via 5’ capping, splicing, and 3’ end processing and splicing are mostly co-transcriptional in humans [45]. Introns of RNA precursors are removed via two steps of transesterification reaction, catalyzed by the spliceosome, which includes five small nuclear RNAs (U1, U2, U4/U6, U5) and many protein factors, such as U2AF2/U2AF65 and SRRM1/SRM160[46,47]. Our results show that spliceosome-related proteins, such as U2AF2, SRRM1, have no significant effect on viral polymerase activity. Of course, the roles of other spliceosome-related factors in viral replication remain to be further elucidated. In contrast, the TREX complex, a major pathway for nuclear mRNA export, which contains a THO subcomplex (THOC1/HPR1, THOC2/hTHO2, THOC3/hTEX1, THOC5/fSAP79, THOC6/fSAP35, THOC4/ALY, CIP29 and THOC7/fSAP24), the RNA helicases (DDX39B/UAP56/BAT1), RNA export adaptors (NXF1/TAP), and co-adaptors (SR proteins), is involved in the regulation of polymerase activity, especially when co-expressed with DDX39B or DDX39A, their inhibitory effects on polymerase activity were more severe. Importantly, our results show that the TREX complex subunits THOC1, THOC4 and CIP29, but not other subunits, are recruited to the RNA-dependent DDX39B-NP complex in an ATP-dependent manner during influenza virus infection, which implies DDX39B and DDX39A, RNA-dependent ATPases, recruit and assemble the TREX complex, a larger active dynamic complex, like a drive motor, which is involved in multiple processes of mRNA processing, such as nucleocytoplasmic transport of viral mRNA. The assembly of the TREX complex is dynamically associated with the ATPase cycle of DDX39B and DDX39A, that is, free DDX39B and DDX39A, leaving the TREX complex when recruiting NXF1 in the late stage of mRNA transport, rely on ATP to combine with THOC1, THOC4, and CIP29 to form small active complexes, paving the way for the next round of mRNA metabolism, which may be requisitioned by influenza virus, due to the phenomenon that a portion of NP proteins are maintained as a monomer for selectively oligomerize into replication complexes is important during the viral life cycle. However, excess TREX-NP complexes interfere with the normal oligomerization state of NP depending on the ratio between the viral and host proteins (Fig 8).

In conclusion, we present a new perspective on the regulation of viral replication by the TREX-NP complex, which fills a cognitive gap in the involvement of the TREX complex in viral replication, except for the mRNA transport process.

## Materials and methods

### Viruses and cells

Human embryonic kidney (HEK293T) cells were cultured in Dulbecco’s Modified Eagle’s medium (DMEM; Gibco) containing 10% fetal bovine serum (FBS; Gibco), 0.2% NaHCO_3_, 100 μg/ml streptomycin, and 100 IU/ml penicillin (Gibco) at 37°C with 5% CO_2_. Madin-Darby Canine Kidney (MDCK) cells were grown in DMEM containing 10% newborn calf serum (NCS; Sigma). Adenocarcinomic human alveolar epithelial (A549) cells and chicken embryo fibroblast (DF-1) cells were grown in DMEM containing 10% NCS. Influenza A virus A/WSN/1933-PB2-627K (H1N1), A/WSN/1933-PB2-627E (a mutant WSN virus encoding PB2 627E), and reassortant viruses WSN-H7 (H1N1, PB2 627K), WSN-H7 (H1N1, PB2 627E) were generated by reverse genetics and inoculated into 10-day-old specific-pathogen-free (SPF) chicken embryos for virus propagation [48]. Viral titers were measured by plaque assay in MDCK cells.

### Plasmid construction

Restriction enzymes were purchased from NEB Inc. (Massachusetts, USA). Primer oligonucleotides were commercially synthesized by GENERAL BIOL (Nanjing, China). Cellular RNA from HEK293T cells, A549 cells and DF-1 cells was extracted by lysing the cells with TRIzol reagent (R0016, Beyotime). The RNA was reverse transcribed into single-stranded DNA using RevertAid First Strand cDNA Synthesis Kit (K1622; Thermo Fisher). Full-length DNA fragments encoding DExD/H-box protein family members, RNA-binding proteins and mitochondrial anchor proteins were amplified by PCR and cloned into the protein expression vector pCAGGS [49] To facilitate western blot detection, HA-, MYC-, or FLAG-tag was added to some gene fragments. For coimmunoprecipitation analysis, the coding regions of the viral PB2, PB1, PA, NP, M1, M2, NS1 and NS2 proteins from influenza virus A/WSN/33 (H1N1) or A/Anhui/1/2013 (H7N9) were cloned into the expression vector pCAGGS in-frame with a FLAG-tag sequence at either the 5’ or 3’ end. Plasmids encoding mutant proteins were constructed by using a site-directed mutagenesis kit (Beyotime, China). All plasmid constructs were verified by Sanger sequencing. All primers and sequences used in this study are listed in S3 Table in the supplemental material.

### Western blot and antibodies

Cells were washed three times with cold phosphate-buffered saline (PBS) and lysed in cold lysis buffer (1% Triton X-100, 1 mM phenylmethylsulfonyl difluoride [PMSF] in PBS) for 30 min. Lysates were clarified by centrifugation at 12,000 × g for 10 min. Proteins in the lysates were separated by SDS-PAGE, transferred to nitrocellulose membranes (10600001; GE Amersham), and then probed with the antibodies indicated in the figure legends. Finally, the membranes were incubated with enhanced chemiluminescence (ECL) reagents (Vazyme, China), and the signals were analyzed using an Amersham Imager 600 CCD-based chemiluminescent analyzer (GE Healthcare). Proteins were detected with the following antibodies: anti-GAPDH (10494-1-AP; proteintech); anti-PB2, anti-PB1, anti-PA and anti-NP antibodies (kindly provided by Dr. Chengjun Li, Harbin Veterinary Research Institute, The Chinese Academy of Agricultural Sciences); anti-DDX39B (14798-1-AP; proteintech)l; anti-DDX39A (11723-1-AP; proteintech); anti-CIP29 (15798-1-AP; proteintech)l; anti-THOC1 (10920-1-AP; proteintech); anti-THOC4 (16690-1-AP; proteintech); anti-FLAG MAb (F1804; Sigma-Aldrich); rabbit anti-FLAG PcAb (9121; HUABIO); anti-MYC MAb (60003-2-Ig; proteintech); and anti-HA MAb (H9658; Sigma-Aldrich).

### Co-Immunoprecipitation (Co-IP)

Transiently transfected cells were washed twice with PBS and lysed in NP-40 lysis buffer (P0013F; Beyotime) supplemented with protease inhibitor (P1005; Beyotime). Whole cell lysate was firstly precleared with protein A/G slurry (sc-2003; Santa Cruz) as follows: After centrifugation at 1,000 × *g* for 5 min at 4°C, supernatant was incubated with 1 μg of mouse anti-FLAG MAb, anti-HA MAb or anti-MYC MAb and 50 μl of protein A/G slurry (sc-2003; Santa Cruz) and incubated with rotation for 4 h at 4°C. Immunoprecipitated samples collected by centrifugation were washed with NP-40 lysis buffer four times. The final pellet was dissolved in 4 × SDS loading buffer (P1016; solarbio) for SDS-PAGE and Western blotting. Immunoprecipitations and the whole-cell lysates were probed with mouse anti-FLAG MAb, rabbit anti-FLAG PcAb and mouse anti-HA MAb or antibodies indicated in the figure legends. HRP-labeled goat anti-mouse light chain as secondary antibody for eliminating heavy chain interference.

### Viral polymerase-minigenome assay

To measure the effect of various cellular factors on influenza polymerase activity, HEK293T cells were transfected in 24-well plates with pCAGGS plasmids encoding the PB2 (100 ng), PB1 (100 ng), PA (100 ng) and NP (100 ng) proteins of human influenza virus H7N9 (A/Anhui/1/2013), H1N1(A/WSN/1933) or avian H9N2 (A/chicken/Anhui/LH99/2017), together with 50 ng species-specific minigenome reporter plasmids (PolI-Luc), 50 ng Renilla luciferase expression plasmids (RL-TK) as an internal control, and plasmids (250 ng) encoding various cellular factors, using Lipofectamine 2000 transfection reagent (Invitrogen) or ExFect transfection reagent (Vazyme) according to manufacturers’ instructions. Cells were incubated at 37°C or 33°C. 48 h after transfection, cells were lysed with 100 μl of passive lysis buffer (Promega), and firefly and Renilla luciferase bioluminescence was measured using a Dual-luciferase system (Promega). To analyze the activity of the influenza viral polymerase in avian DF-1 cells, minigenome reporter plasmids containing the chicken RNA polymerase I promoter (PolIck-Luc) were constructed. The expression levels of PB2, NP or cellular factors in different groups were assessed by western blotting using specific antibodies.

### shRNA-mediated silencing

Silencing was achieved by lentivirus delivery of shRNA encoding transgenes. Lentiviral vectors were generated by using the TRC1.5-pLKO.1-puro plasmid (MISSION Sigma-Aldrich) containing the shRNA sequence and puromycin selection gene. The pLKO.1 vector containing shRNA sequences targeting specific genes along with lentiviral packaging vectors, psPAX2 and pMD2.G, were transfected into HEK293T cells with Lipofectamine 3000 (Invitrogen). After 48 h, the lentiviral supernatants were collected and used to infect HEK293T cells at a multiplicity of infection of 3. The infected cells were selected with puromycin (1 μg/ml) for 48 h and examined for knockdown efficiency by immunoblotting. shRNA sequences for target genes were as follows: DDX39B-hu shRNA (target sequence, 5’-GAAGAAGAACTGCCCGCATAT-3’), DDX39A-hu shRNA (target sequence, 5’-GCTGGAGTTTAACCAGGTGAT-3’), CIP29-hu shRNA (target sequence, 5’-GAGACCAAGGGAATAAAGCAA-3’), THOC4-hu shRNA (target sequence, 5’-GCCGATATTCAGGAACTCTTT-3’), THOC1-hu shRNA (target sequence, 5’-GATACCAAACCTACGAGAATA-3’).

### siRNA-mediated silencing

HEK293T cells were transfected with 20 nM of target genes siRNA or negative control (NC.si) using Lipofectamine 2000 (Invitrogen) in 24-well plates, according to manufacturer’s instructions. Twenty-four hours later, cells were transfected with polymerase and minigenome constructs or infected with the specified influenza virus. siRNAs and negative control (NC.si) were purchased from Genepharma (Shanghai, China). siRNAs for target genes were as follows: siDDX39B: 5’-AAGGGCUUGGCUAUCACAUUU-3’, siDDX39A: 5’-AAAGGCCUAGCCAUCACUUUU-3’, NC.si: 5’-UUCUCCGAACGUGUCACGUTT-3’.

### Quantitative real-time PCR (qPCR)

Influenza virus-infected cells were washed three times with PBS, and total RNA was extracted by using TRIzol reagent (R0016, Beyotime). Total RNA (1 μg) was subjected to first-strand cDNA synthesis with specific primers (vRNAtag-PB2 F:GGCCGTCATGGTGGCGAATGATGCGTGACGTACTGGGAAC) or oligo(dT)_20_ using HiScript II 1st Strand cDNA Synthesis Kit (Vazyme, China). Quantitative real-time PCR was then performed using the cDNAs and gene-specific primer pairs with the AceQ qPCR SYBR Green Master Mix (Vazyme, China) in a Roche LightCycler 96, according to the manufacturer’s instructions using the following cycling program: 95°C for 5 mins, 40 cycles at 95°C for 10 seconds, and 60°C for 30 seconds. The 2^(-ΔΔCt) method was used to determine the relative mRNA or vRNA levels of candidate genes. Gene specific primer pairs were as follows: vRNA PB2 (5’-GGCCGTCATGGTGGCGAAT-3’ and 5’-CCCCTCAATACCGCAGATTCC-3’), mRNA PB2 (5’-GATGCGTGACGTACTGGGAAC-3’ and 5’-CCCCTCAATACCGCAGATTCC-3’), GAPDH (5’-GCCAAGGCTGTGGGCAAGG-3’ and 5’-GGAGGAGTGGGTGTCGCTG-3’), U6 (5’-CTCGCTTCGGCAGCACA-3’ and 5’-AACGCTTCACGAATTTGCGT-3’).

### Sample preparation and mass spectrometry

HEK293T cells were transfected in triplicate wells (about 80% confluent, 6-well plates) with 600 ng each of pCAGGS plasmids encoding a PB2 mutant [PB2_627_E, PB2_627_K, PB2_627_ domain del (lacking amino acids 535–667 of PB2), PB2 627 CON (possessing stop codons at amino acid positions 49 and 64)] and plasmids encoding PB1, PA, and NP proteins, together with 600 ng of the minigenome reporter plasmids (PolI-Luc), using Lipofectamine 2000 transfection reagent (Invitrogen) according to manufacturers’ instructions. Similarly, the same amounts of polymerase and NP protein expression plasmids, together with PolIck-Luc plasmids were co-transfected into three wells of DF-1 cells (about 90% confluence, 6-well plates). The immunoprecipitates were then subjected to mass spectrometry as follows: First, the protein samples were subjected to reductive alkylation and enzymatic hydrolysis. The peptides produced by enzymatic hydrolysis were desalted with a C18 column, and the desalted peptides were drained and then dissolved in 15 μl Loading Buffer (0.1% formic acid, 3% acetonitrile). The peptides were analyzed on the LC-MS/MS (AB Sciex TripleTOF 5600-plus) instrument, and the original data were directly submitted to the Proteinpilot software connected to the AB SCIEX Triple TOF 5600 plus mass spectrometer for database search. After removing common contaminating proteins such as keratin, antibody protein, and serum albumin, peptides that were detected at least once with a confidence level over 95% were included in the further analysis. Detailed information of all proteins identified in these samples is listed in S1 Table in the supplementary material.

### Gene annotation and enrichment analysis

Analysis was performed using Metascape (http://metascape.org), a free, user-friendly analysis tool for gene annotation and analysis [50]. In this study, Metascape was used to conduct ID conversion of input gene identifiers into Entrez gene IDs, annotations for the gene list using GO Biological Processes and KEGG and functional enrichment analysis. Only terms with P-value < 0.01, minimum count of 3, and enrichment factor of > 1.5 were considered as significant. We firstly identified all statistically enriched terms (GO/KEGG terms, canonical pathways, hall mark gene sets, etc.), accumulative hypergeometric p-values and enrichment factors were calculated and used for filtering. Remaining significant terms were then hierarchically clustered into a tree based on Kappa-statistical similarities among their gene memberships. Then 0.3 kappa score was applied as the threshold to cast the tree into term clusters. Protein–protein interaction enrichment analysis was performed using the following databases: BioGrid, InWeb_IM, and OmniPath. Further, resultant network contains the subset of proteins that form physical interactions with at least one other member in the list. If the network contains between 3 and 500 proteins, the Molecular Complex Detection (MCODE) algorithm has been applied to identify densely connected network components. The MCODE networks identified for individual gene lists have been gathered. Pathway and process enrichment analysis has been applied to each MCODE component independently, and the three best-scoring terms by p-value have been retained as the functional description of the corresponding components. Networks were visualized with Cytoscape (ver. 3.7.2).

### Immunofluorescence

HEK293T cells were seeded on glass coverslips and transfected with the indicated plasmids. At the indicated timepoints, cells were fixed with 4% paraformaldehyde and permeabilized with a solution of PBS containing 0.2% Triton X-100 for 10 minutes (Sigma). Then, the cells were washed and stained for 1 h with primary antibody followed by staining with fluorescently-labelled secondary antibody (goat anti-mouse FITC-labeled secondary antibody, 172–1806, KPL; goat anti-mouse DyLight 405-labeled secondary antibody, A0609, Beyotime; goat anti-rabbit FITC-labeled secondary antibody, BF05002, Biodragon; goat anti-rabbit Alexa Fluor 546-labeled secondary antibody, A21085, Invitrogen) in a 5% bovine serum albumin (BSA; Thermo Scientific) solution in PBS. Cells were then stained with 4,6-diamidino-2-phenylindole (DAPI) for 10 min. Finally, images were acquired using confocal microscopy (Nikon, Japan) equipped with micro-objective (Plan Apo 60×/1.40, oil immersion, Nikon, Japan) and Microscope eyepiece (CFI, 10×/22, Nikon) [51].

### RNA-fluorescence in situ hybridization (FISH)

FISH assays were performed using Fluorescence In Situ Hybridization Kit (GenePharma, Shanghai, China) according to the instructions. Briefly, HEK293T cells were seeded on glass coverslips and infected with the indicated viruses. The cells were fixed with 4% paraformaldehyde at 12 h after infection and then permeabilized with 0.1% Triton X-100. After washing with 1x SSC (Saline Sodium Citrate Buffer) /50% formamide, the cells were incubated with the probe overnight at 42°C. DAPI was used for nuclear staining. The following 5’ cy3-labeled FISH probes to the mRNA of A/Anhui/1/2013 (H7N9) NP were used: 5’-GCTTGTCGCCAAATTCTCCT-3’, 5’-CCATCATTGCCCTTTGTGCT-3’, 5’-CTAAGGTCCTCAAACGCTGC-3’ (all synthesized by Genepharma, Shanghai, China).

### Isolation of Nuclear and Cytoplasmic RNAs

Nuclear and cytoplasmic RNA were isolated separately using the Cytoplasmic & Nuclear RNA purification kit (Norgen Biotek, Thorold, ON, USA) according to the manufacturer’s protocol.

### Plaque assays

The infectious titers of influenza viruses were determined by plaque assays according to the procedures described previously. Briefly, viruses were serially 10-fold diluted and inoculated onto MDCK cell monolayers. After incubation at 37°C for 60 min, the cells were overlaid with DMEM containing 1% SeaPlaque agarose (Lonza) and 1 μg/ml TPCK-trypsin and incubated at 37°C. At 2 days post infection (d.p.i.), Check for plaque formation.

### Statistical analysis

The data are shown as mean ± SD, and unless otherwise indicated, all the presented data are representative results of at least three independent repeats. Statistical analysis was performed with Prism 8 (GraphPad), and the statistics were analyzed by two-tailed Student’s t test or one-way or two-way ANOVA as indicated. Differences considered to be significant at P < 0.05 are indicated by *, those at P < 0.01 are indicated by **, those at P < 0.001 are indicated by ***, those at P < 0.0001 are indicated by ****, ns, no significance.

## Supporting information

S1 Fig Identification of differential interaction factors with influenza virus polymerase complex PB2_627_K and PB2_627_E. (A) Schematic of vRNP complex construction. Briefly, PB2 mutants (PB2_627_E, PB2_627_K, PB2_627_ domain del, PB2_627_ CON) were separately co-transfected with PB1, PA, NP, and PolI-Luc into HEK293T cells. Similarly, polymerase complex expression plasmids and PolIck-Luc were co-transfected into DF-1 cells. (B) Schematic of the construction of different PB2 mutants. (C) The four PB2 mutants in the above were separately co-transfected with PB1, PA and NP, together with PolI-Luc and RL-TK into HEK293T cells. Cells were cultured at 37°C or 33°C, 48h after transfection, the polymerase activity was detected. Statistical differences between groups are labeled according to a two-way ANOVA. Each treatment was repeated three times in parallel. The results are presented as means ± standard deviations. ****, P < 0.0001.
(TIF)

S2 Fig Subcellular localization and expression levels of DDX39B and DDX39A during infection. (A) PB2_627_E, PB1, PA and NP from avian influenza virus H7N9 (A/Anhui/1/2013), PolI-Luc and RL-TK, together with HA-DDX39B, HA-DDX39A, DDX39B-GFP, DDX39A-mCherry or empty pCAGGS vectors were co-transfected into 293T cells, 48h after transfection, the polymerase activity was detected. (B and C) DDX39B-mCherry (B) or DDX39A-mCherry (C) fusion protein expression plasmids were transfected into HEK293T cells, 24 hours after transfection, cells were infected with recombinant influenza virus WSN-H7 (627E) at MOI = 1. At 4h, 8h, 12h after infection, the cells were then fixed, permeabilized, and stained with NP antibody followed by immunostaining with FITC-labeled goat anti-mouse secondary antibody. And cells were stained with the 4, 6-diamidino-2-phenylindole (DAPI) and were examined via fluorescence microscopy. Statistical differences between groups are labeled according to a one-way ANOVA followed by a Dunnett’s test. Each treatment was repeated three times in parallel. The results are presented as means ± standard deviations. ****, P < 0.0001; ns, no significance.
(TIF)

S3 Fig Alignment of amino acids of DDX39B or DDX39A proteins. The known or supposed function of the respective motifs are indicated by shaded boxes. All sequences were aligned with ClustalW and mapped online program, ESPript server [52].
(TIF)

S4 Fig Detection of the interactions of human DDX39B, DDX39A and avian DDX39B with M1, M2 or NS2 protein. (A) PB2_627_E, PB1, PA and NP from avian influenza virus H7N9 (A/Anhui/1/2013), poll-Luc, RL-TK and HA-DDX39A, together with M1, M2, NS1, NS2 of influenza virus WSN/1933 or empty pCAGGS vectors were co-transfected into HEK293T cells, 48h after transfection, the polymerase activity was detected. (B, C and D) HEK293T cells were co-transfected with HA-DDX39B, HA-DDX39A or HA-DDX39B-CK and FLAG-M1 (B), FLAG-M2 (C), FLAG-NS2 (D) of influenza virus A/WSN/1933 (H1N1), at 48h after transfection, the cells were lysed, followed by co-IP with anti-HA mouse Mab and western blotting using anti-Flag mouse Mab and anti-HA mouse MAb. Statistical differences between groups are labeled according to a one-way ANOVA followed by a Dunnett’s test. Each treatment was repeated three times in parallel. The results are presented as means ± standard deviations. ****, P < 0.0001.
(TIF)

S5 Fig The effects of splicing factors on regulating influenza virus polymerase activity. (A) HEK293T cells were co-transfected with FLAG-DDX39B and HA-SRSF7, HA-U2AF2 or empty pCAGGS vectors, at 48h after transfection, the cells were lysed, followed by co-IP with anti-FLAG mouse Mab and western blotting using anti-Flag mouse Mab and anti-HA mouse MAb. (B) PB2_627_E, PB1, PA and NP protein expression plasmids, poll-Luc, RL-TK and DDX39B together with protein expression plasmids encoding SRSF7, U2AF2, SRRM1 or empty pCAGGS vectors were co-transfected into HEK293T cells, 48h after transfection, and the polymerase activity was detected. (C) PB2_627_E, PB1, PA and NP from avian influenza virus H7N9 (A/Anhui/1/2013), poll-Luc, RL-TK, together with HA-DDX39B, THOC1, THOC2, THOC3, THOC4, THOC5, THOC6, THOC7, CIP29 or empty pCAGGS vectors were co-transfected into HEK293T cells, 48h after transfection, the polymerase activity was detected. (D) PB2_627_E, PB1, PA and NP from avian influenza virus H7N9 (A/Anhui/1/2013), poll-Luc, RL-TK and HA-DDX39A, together with THOC1, THOC2, THOC3, THOC4, THOC5, THOC6, THOC7, CIP29 or empty pCAGGS vectors were co-transfected into HEK293T cells, 48h after transfection, the polymerase activity was detected. Statistical differences between groups are labeled according to a one-way ANOVA followed by a Dunnett’s test. Each treatment was repeated three times in parallel. The results are presented as means ± standard deviations. (E) Validation of shRNA-mediated knockdown of THOC1, THOC4 or CIP29 cell lines. (F) PB2_627_E, PB1, PA and NP from avian influenza virus H7N9 (A/Anhui/1/2013), poll-Luc, RL-TK, together with HA-DDX39B or empty pCAGGS vectors were co-transfected into THOC1, THOC4 or CIP29 knockdown HEK293T cell lines, 48h after transfection, the polymerase activity was detected. *, P < 0.05; **, P < 0.01; ***, P < 0.001; ****, P < 0.0001; ns, no significance.
(TIF)

S6 Fig DDX39B/DDX39A recruits TREX complex subunits THOC1, THOC4 and CIP29 to form a complex. (A, B and C) HEK293T cells were co-transfected with MYC-THOC1 (A), HA-THOC4 (B) or MYC-CIP29 (C) and FLAG-DDX39B (WT), FLAG-DDX39B (95A) or empty pCAGGS vectors, at 48h after transfection, the cells were lysed, followed by co-IP with anti-FLAG mouse Mab and western blotting using the antibodies specified in the legend. (D and E) DDX39B-GFP (D) or DDX39A-mCherry (E) together with NP were transfected into HEK293T cells, 24 hours after transfection, the cells were fixed, permeabilized, and stained with anti-NP mouse Mab together with anti-CIP29, anti-THOC1 or anti-THOC4 rabbit PcAb, followed by immunostaining with goat anti-mouse DyLight 405-labeled secondary antibody and goat anti-rabbit FITC-labeled secondary antibody or goat anti-rabbit Alexa Fluor 546-labeled secondary antibody. Images were acquired using confocal microscopy.
(TIF)

S1 Table Identified peptides and proteins via LC-MS. (XLSX)

S2 Table Gene ontology and pathway enrichment analysis. (XLSX)

S3 Table Primers and sequences used in this article. (XLSX)
