# Supplementary figures and images for: TREX (transcription/export)-NP complex exerts a dual effect on regulating polymerase activity and replication of influenza A virus
Source: PLoS Pathog. 2022 Sep 9;18(9):e1010835. doi: 10.1371/journal.ppat.1010835 (PMC9491529; doi:10.1371/journal.ppat.1010835)

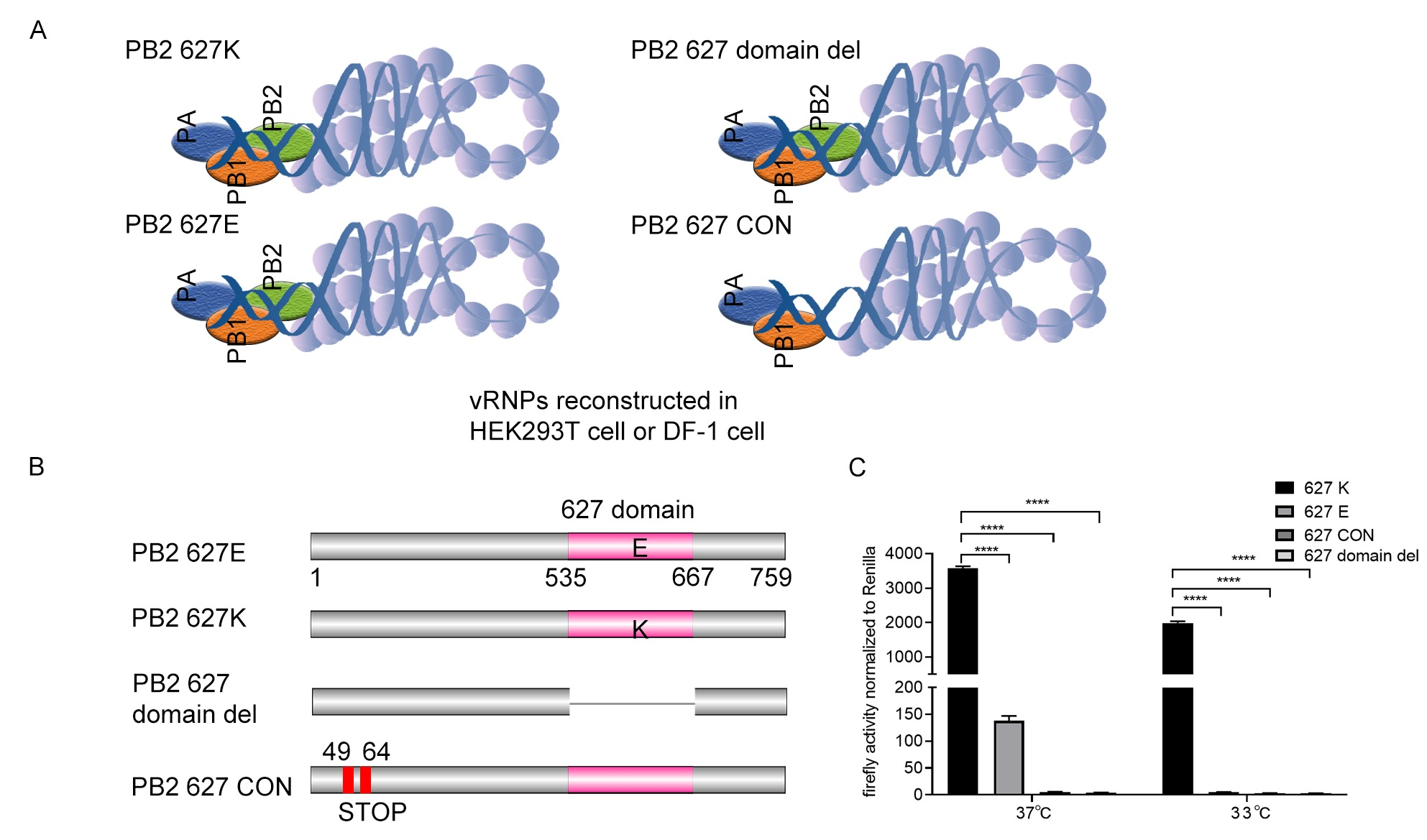

Supplement: S1 Fig — (A) Schematic of vRNP complex construction. Briefly, PB2 mutants (PB2627E, PB2627K, PB2627 domain del, PB2627 CON) were separately co-transfected with PB1, PA, NP, and PolI-Luc into HEK293T cells. Similarly, polymerase complex expression plasmids and PolIck-Luc were co-transfected into DF-1 cells. (B) Schematic of the construction of different PB2 mutants. (C) The four PB2 mutants in the above were separately co-transfected with PB1, PA and NP, together with PolI-Luc and RL-TK into HEK293T cells. Cells were cultured at 37°C or 33°C, 48h after transfection, the polymerase activity was detected. Statistical differences between groups are labeled according to a two-way ANOVA. Each treatment was repeated three times in parallel. The results are presented as means ± standard deviations. ****, P < 0.0001. (TIF) [file ppat.1010835.s001.tif]

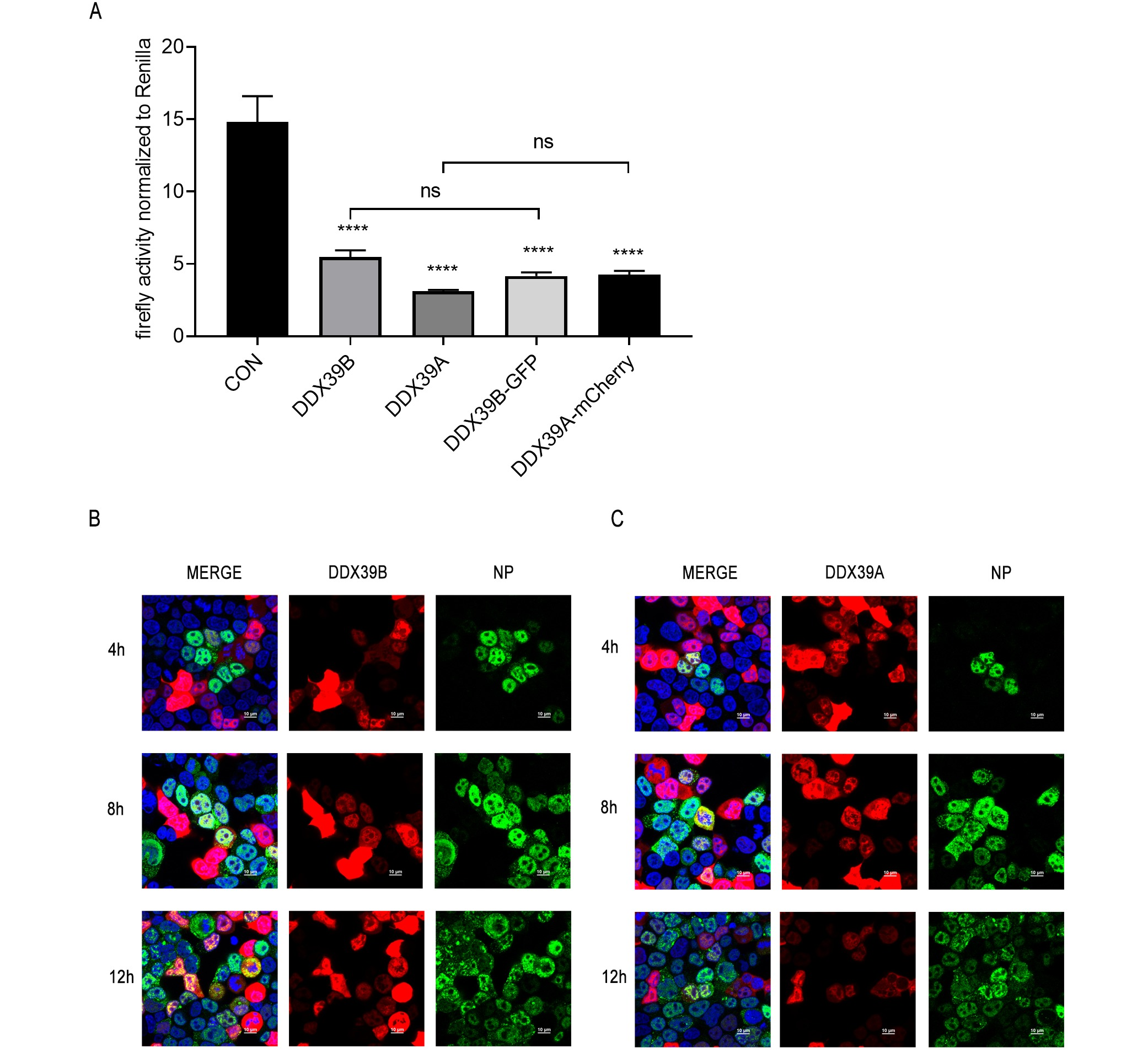

Supplement: S2 Fig — (A) PB2627E, PB1, PA and NP from avian influenza virus H7N9 (A/Anhui/1/2013), PolI-Luc and RL-TK, together with HA-DDX39B, HA-DDX39A, DDX39B-GFP, DDX39A-mCherry or empty pCAGGS vectors were co-transfected into 293T cells, 48h after transfection, the polymerase activity was detected. (B and C) DDX39B-mCherry (B) or DDX39A-mCherry (C) fusion protein expression plasmids were transfected into HEK293T cells, 24 hours after transfection, cells were infected with recombinant influenza virus WSN-H7 (627E) at MOI = 1. At 4h, 8h, 12h after infection, the cells were then fixed, permeabilized, and stained with NP antibody followed by immunostaining with FITC-labeled goat anti-mouse secondary antibody. And cells were stained with the 4, 6-diamidino-2-phenylindole (DAPI) and were examined via fluorescence microscopy. Statistical differences between groups are labeled according to a one-way ANOVA followed by a Dunnett’s test. Each treatment was repeated three times in parallel. The results are presented as means ± standard deviations. ****, P < 0.0001; ns, no significance. (TIF) [file ppat.1010835.s002.tif]

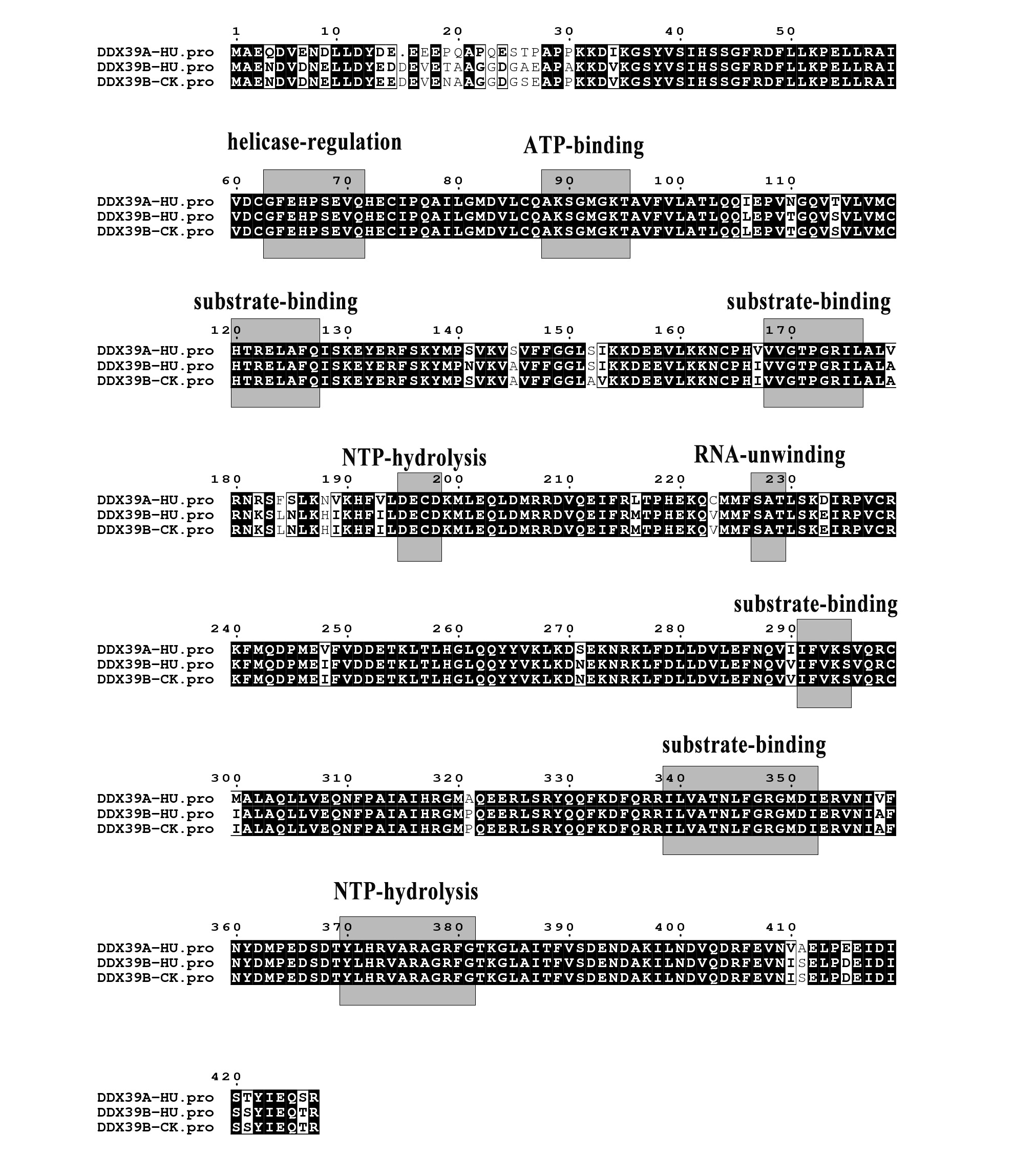

Supplement: S3 Fig — The known or supposed function of the respective motifs are indicated by shaded boxes. All sequences were aligned with ClustalW and mapped online program, ESPript server [52]. (TIF) [file ppat.1010835.s003.tif]

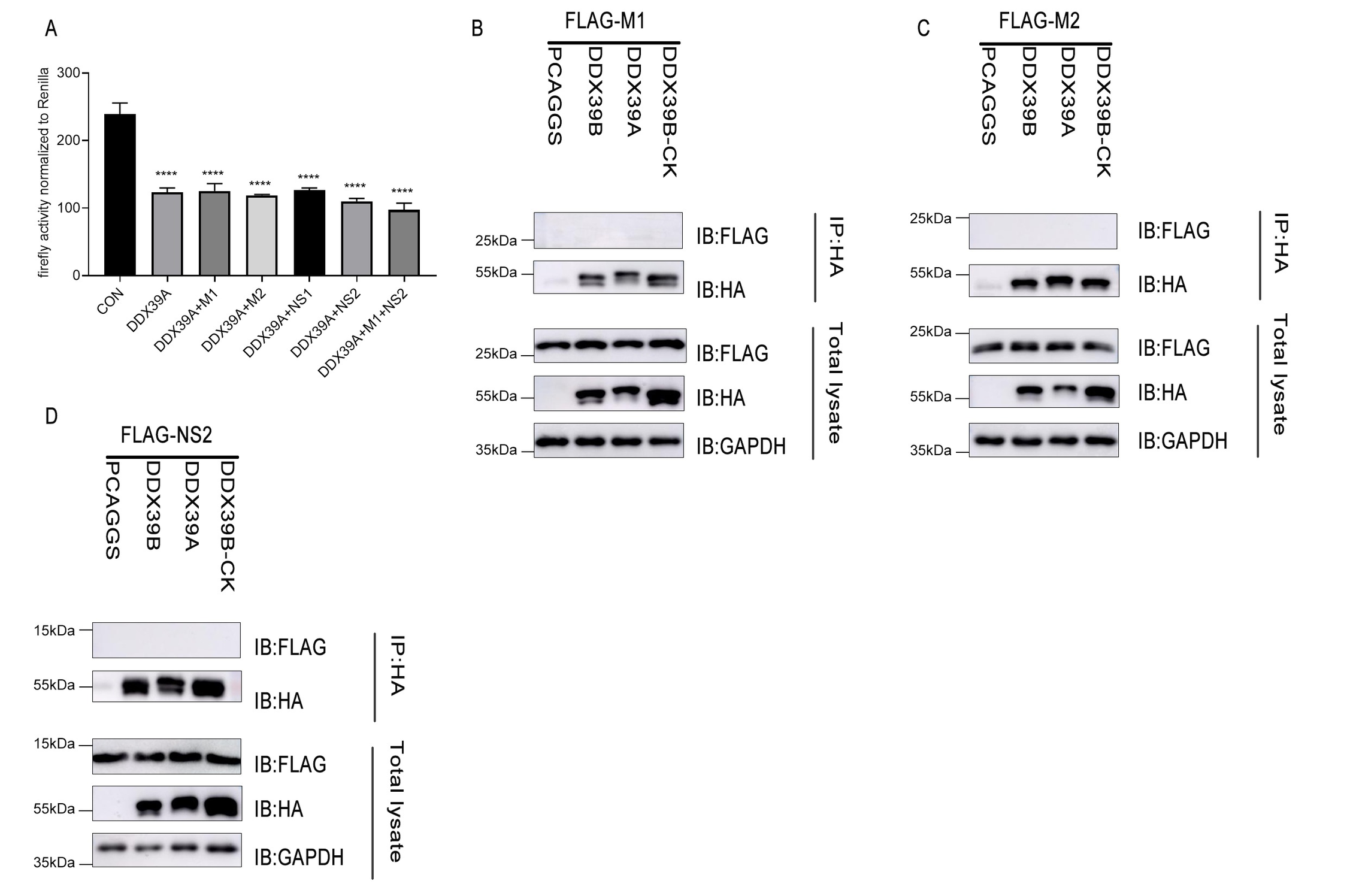

Supplement: S4 Fig — (A) PB2627E, PB1, PA and NP from avian influenza virus H7N9 (A/Anhui/1/2013), poll-Luc, RL-TK and HA-DDX39A, together with M1, M2, NS1, NS2 of influenza virus WSN/1933 or empty pCAGGS vectors were co-transfected into HEK293T cells, 48h after transfection, the polymerase activity was detected. (B, C and D) HEK293T cells were co-transfected with HA-DDX39B, HA-DDX39A or HA-DDX39B-CK and FLAG-M1 (B), FLAG-M2 (C), FLAG-NS2 (D) of influenza virus A/WSN/1933 (H1N1), at 48h after transfection, the cells were lysed, followed by co-IP with anti-HA mouse Mab and western blotting using anti-Flag mouse Mab and anti-HA mouse MAb. Statistical differences between groups are labeled according to a one-way ANOVA followed by a Dunnett’s test. Each treatment was repeated three times in parallel. The results are presented as means ± standard deviations. ****, P < 0.0001. (TIF) [file ppat.1010835.s004.tif]

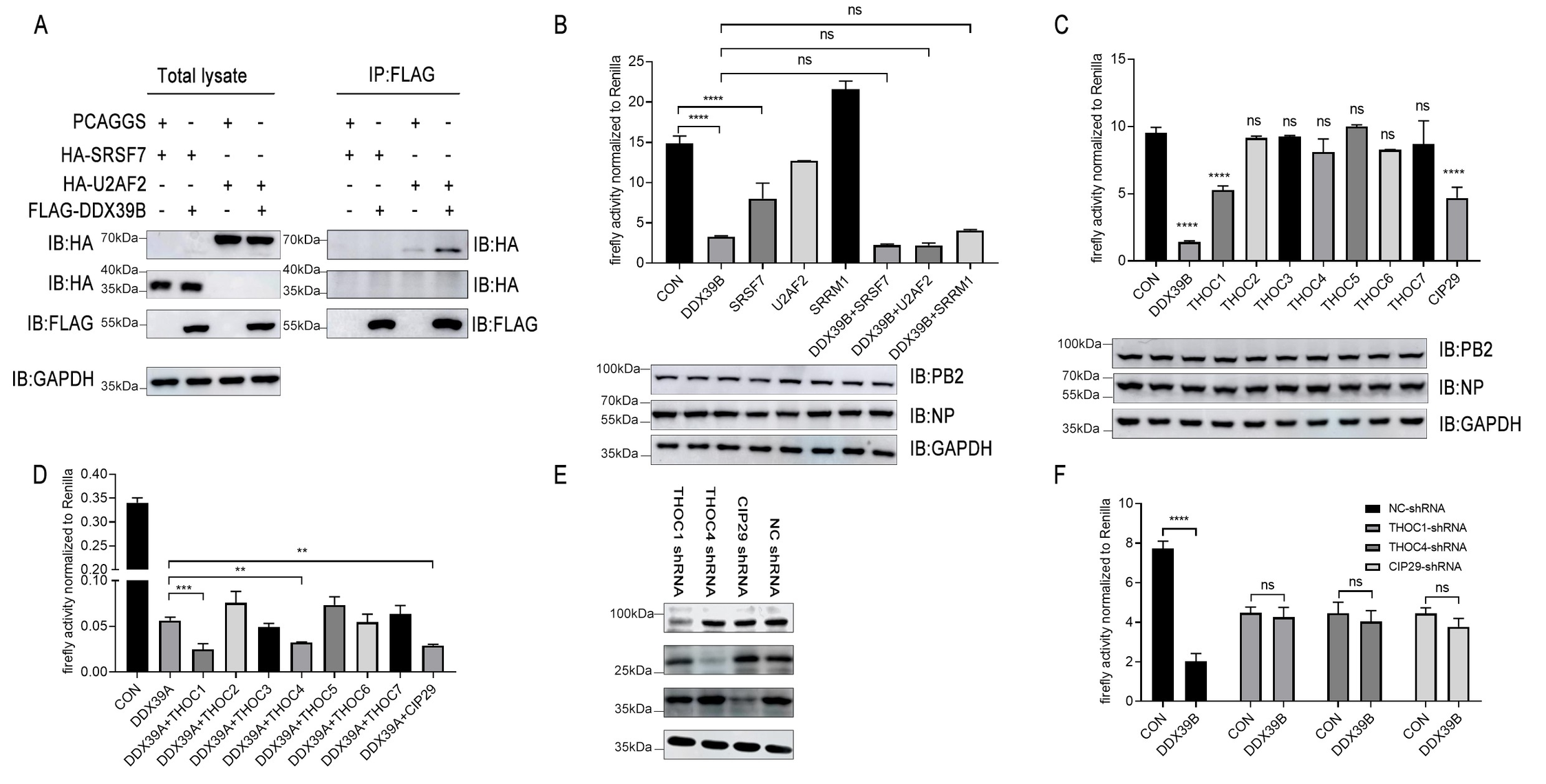

Supplement: S5 Fig — (A) HEK293T cells were co-transfected with FLAG-DDX39B and HA-SRSF7, HA-U2AF2 or empty pCAGGS vectors, at 48h after transfection, the cells were lysed, followed by co-IP with anti-FLAG mouse Mab and western blotting using anti-Flag mouse Mab and anti-HA mouse MAb. (B) PB2627E, PB1, PA and NP protein expression plasmids, poll-Luc, RL-TK and DDX39B together with protein expression plasmids encoding SRSF7, U2AF2, SRRM1 or empty pCAGGS vectors were co-transfected into HEK293T cells, 48h after transfection, and the polymerase activity was detected. (C) PB2627E, PB1, PA and NP from avian influenza virus H7N9 (A/Anhui/1/2013), poll-Luc, RL-TK, together with HA-DDX39B, THOC1, THOC2, THOC3, THOC4, THOC5, THOC6, THOC7, CIP29 or empty pCAGGS vectors were co-transfected into HEK293T cells, 48h after transfection, the polymerase activity was detected. (D) PB2627E, PB1, PA and NP from avian influenza virus H7N9 (A/Anhui/1/2013), poll-Luc, RL-TK and HA-DDX39A, together with THOC1, THOC2, THOC3, THOC4, THOC5, THOC6, THOC7, CIP29 or empty pCAGGS vectors were co-transfected into HEK293T cells, 48h after transfection, the polymerase activity was detected. Statistical differences between groups are labeled according to a one-way ANOVA followed by a Dunnett’s test. Each treatment was repeated three times in parallel. The results are presented as means ± standard deviations. (E) Validation of shRNA-mediated knockdown of THOC1, THOC4 or CIP29 cell lines. (F) PB2627E, PB1, PA and NP from avian influenza virus H7N9 (A/Anhui/1/2013), poll-Luc, RL-TK, together with HA-DDX39B or empty pCAGGS vectors were co-transfected into THOC1, THOC4 or CIP29 knockdown HEK293T cell lines, 48h after transfection, the polymerase activity was detected. *, P < 0.05; **, P < 0.01; ***, P < 0.001; ****, P < 0.0001; ns, no significance. (TIF) [file ppat.1010835.s005.tif]

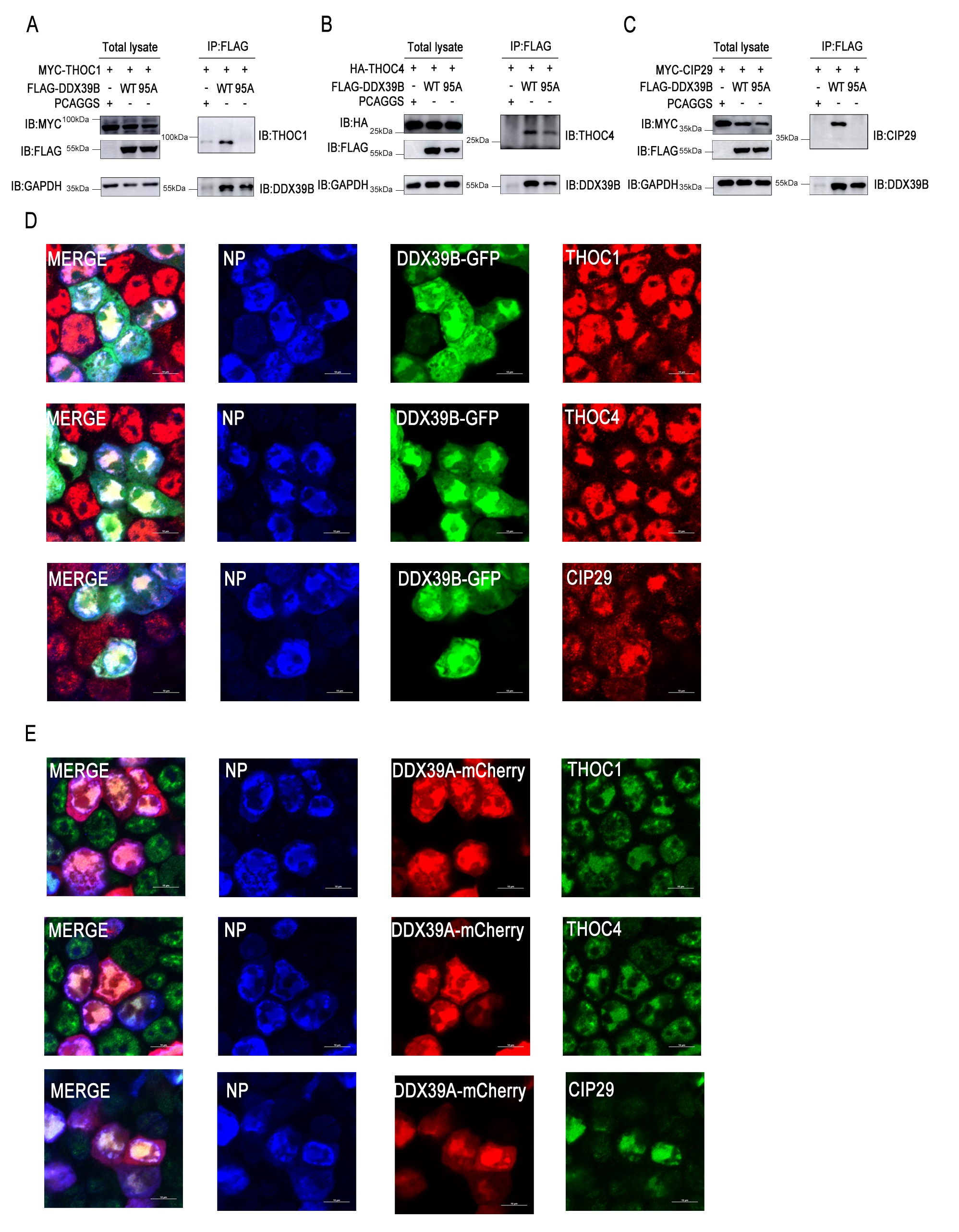

Supplement: S6 Fig — (A, B and C) HEK293T cells were co-transfected with MYC-THOC1 (A), HA-THOC4 (B) or MYC-CIP29 (C) and FLAG-DDX39B (WT), FLAG-DDX39B (95A) or empty pCAGGS vectors, at 48h after transfection, the cells were lysed, followed by co-IP with anti-FLAG mouse Mab and western blotting using the antibodies specified in the legend. (D and E) DDX39B-GFP (D) or DDX39A-mCherry (E) together with NP were transfected into HEK293T cells, 24 hours after transfection, the cells were fixed, permeabilized, and stained with anti-NP mouse Mab together with anti-CIP29, anti-THOC1 or anti-THOC4 rabbit PcAb, followed by immunostaining with goat anti-mouse DyLight 405-labeled secondary antibody and goat anti-rabbit FITC-labeled secondary antibody or goat anti-rabbit Alexa Fluor 546-labeled secondary antibody. Images were acquired using confocal microscopy. (TIF) [file ppat.1010835.s006.tif]
